# Amyloidogenic motifs revealed by n-gram analysis

**DOI:** 10.1038/s41598-017-13210-9

**Published:** 2017-10-11

**Authors:** Michał Burdukiewicz, Piotr Sobczyk, Stefan Rödiger, Anna Duda-Madej, Paweł Mackiewicz, Małgorzata Kotulska

**Affiliations:** 1 Department of Genomics, University of Wrocław, Wrocław, Poland; 2 Faculty of Pure and Applied Mathematics, Wrocław University of Science and Technology, Wrocław, Poland; 3 Institute of Biotechnology, Brandenburg University of Technology Cottbus-Senftenberg, Senftenberg, Germany; 4 Department of Microbiology, Wrocław Medical University, Wrocław, Poland; 5 Faculty of Fundamental Problems of Technology, Department of Biomedical Engineering, Wrocław University of Science and Technology, Wrocław, Poland

## Abstract

Amyloids are proteins associated with several clinical disorders, including Alzheimer’s, and Creutzfeldt-Jakob’s. Despite their diversity, all amyloid proteins can undergo aggregation initiated by short segments called hot spots. To find the patterns defining the hot spots, we trained predictors of amyloidogenicity, using n-grams and random forest classifiers. Since the amyloidogenicity may not depend on the exact sequence of amino acids but on their more general properties, we tested 524,284 reduced amino acid alphabets of different lengths (three to six letters) to find the alphabet providing the best performance in cross-validation. The predictor based on this alphabet, called AmyloGram, was benchmarked against the most popular tools for the detection of amyloid peptides using an external data set and obtained the highest values of performance measures (AUC: 0.90, MCC: 0.63). Our results showed sequential patterns in the amyloids which are strongly correlated with hydrophobicity, a tendency to form *β*-sheets, and lower flexibility of amino acid residues. Among the most informative n-grams of AmyloGram we identified 15 that were previously confirmed experimentally. AmyloGram is available as the web-server: http://smorfland.uni.wroc.pl/shiny/AmyloGram/ and as the R package **AmyloGram**. R scripts and data used to produce the results of this manuscript are available at http://github.com/michbur/AmyloGramAnalysis.

## Introduction

Amyloid aggregates have been observed in tissues of people suffering from neurodegenerative disorders such as Alzheimer’s, Parkinson’s, and Huntington’s diseases and amyotrophic lateral sclerosis, as well as many other conditions^1^. These aggregates were also detected in non-neurological disorders including type 2 diabetes and certain types of cataracts. Cells in tissues with amyloid oligomers exhibit very high mortality, but the exact mechanisms of the cytotoxicity have not been discovered. Amyloids are resistant to activity of proteolytic enzymes and chemical compounds due to the specific and highly ordered structure of their steric zipper. However, some strategies to prevent amyloid formation have been proposed^2^.

Aggregation occurs when a cell environment fosters the partial unfolding of protein chains or their fragmentation in a way that exposes the parts prone to joining with similar protein fragments. The formation of the non-native partially unfolded conformation is required to start the aggregation, presumably by enabling specific intermolecular interactions including electrostatic attraction, hydrogen bonding and hydrophobic contacts^3^.

Then the resulting molecules form oligomers which may grow into larger aggregates. The aggregates may be either unstructured amorphous clusters or highly ordered amyloids that finally form fibrils. Independent of the protein sequence and its original structure, amyloid aggregates always display a common cross-*β* structure^4^. The structure of the steric zipper enables distinction between amyloids and amorphous aggregates using either a variety of microscopic techniques or fluorescence of probes with which they form compounds. Aggregation can also be induced in non-amyloidogenic peptides by conditions such as very high concentration, low pH, high temperature, or oxidative stress.

It is currently believed that short peptide sequences with amyloidogenic properties, called hot spots, are responsible for the aggregation of amyloid proteins. Previous studies have suggested that amyloidogenic fragments may have regular characteristics, not only with regard to averaged physicochemical properties of their amino acids, but also the order of amino acids in the sequence.

It is important to distinguish between amyloidogenic and amyloid (or amyloidic) peptides, because only the former are capable of initiating the process of aggregation. The latter may consist of amyloidogenic hot-spots as well as other regions that are not directly responsible for the onset of aggregation process, although involved in the final aggregate. Several computational approaches have been proposed to model and predict both kinds of regions. Physics- and chemistry-based models used in FoldAmyloid^5^ use the density of the protein contact sites. Other methods, such as PASTA 2.0, AmyloidMutants, or TANGO, perform threading a peptide on an amyloid fiber backbone, followed by determination of its energy and stability^6–9^. Statistical approaches include production of frequency profiles, such as the WALTZ method^10^ and machine learning methods, for example those developed in our group^11,12^. AGGRESCAN3D was proposed to estimate more accurately aggregation propensity by performing 3D structure based analysis^13^.

The aim of our study is to automatically generate thousands of hot spot models, select from them the most appropriate one and gain new insight into the mechanism of amyloidogenicity from its analysis. To do so, we combined n-gram analysis with the reduction of amino acid alphabet.

In bioinformatics, n-grams (k-mers) are continuous or discontinuous sequences of n elements. Employed as a feature extraction method, n-grams are widely used in various analyses of biological sequences. Our choice of n-grams was driven by their highly interpretable nature. This feature is valuable here because we are interested in identification of motifs that are most relevant to amyloidogenic properties of peptides.

Several studies have highlighted that three-dimensional protein structure depends not only on the exact sequence of amino acids but also on their general physicochemical properties. Therefore, a reduced amino acid alphabet (encoding), which represents certain subgroups of amino acids, can still retain the information about the protein folding^14^. Since amyloid aggregates, especially their hot spot regions, have very specific spatial organization, we investigated if these regions can be described by a shorter amino acid alphabet. Hence, we created multiple encodings based on the combinations of various physicochemical properties that might be associated with amyloidogenicity.

To discover amino acid patterns specific for amyloidogenicity, we based our analysis on n-grams drawn from the encoded peptides. The extraction of n-grams allows the detection of more elaborate motifs, but creates very large feature spaces. Then, we used a novel feature selection algorithm, Quick Permutation Test (QuiPT), to select the most informative n-grams.

We used the selected n-grams to train a predictor based on the random forest method^15^ to discriminate between amyloidogenic and non-amyloidogenic peptides. We trained the classifier for several iterations on peptides of varying lengths to identify the optimal number of residues which include the information about the occurrence or absence of a hot spot. In the cross-validation setup, we found the encoding associated with the best-performing classifier and its set of informative n-grams. Finally, we benchmarked our best-performing classifier, AmyloGram, on an external data set against other state-of-the- art software tools for prediction of amyloid or amyloidogenic regions.

## Methods

### Data set

The data used in the study were extracted from the AmyLoad database^16^ and included 421 amyloid peptides and 1044 non-amyloid peptides (1465 sequences in total). Although even bipeptides can form amyloid aggregates^17^, very short sequences are not sufficiently represented in experimentally verified databases. Hexapeptides dominate in the amyloid data sets. They are also regarded as very good representatives of amyloid hot-spots, which are believed to include typically between 4 and 10 amino acids. To create representative data sets for our method, we assumed that a minimum length of fragments is six residues. Sequences shorter than six and longer than 25 amino acid residues (8 and 27 sequences, respectively) were removed from the set because the former were too short to be processed in the devised n-gram analysis framework and the latter were too diversified and rare, hampering a proper analysis. In total, the final data set contained 1430 peptides: 397 amyloid and 1033 non-amyloid sequences (Table 1).

**Table 1:** Characteristics of training and test data sets used in the cross-validation.

Set	Sequence length	Status	Sequences	Hexapeptides
Training	6	Non-amyloid	841	841
		Amyloid	247	247
	6–10	Non-amyloid	964	1412
		Amyloid	312	475
	6–15	Non-amyloid	992	1653
		Amyloid	342	720
Test	6	Non-amyloid	841	841
		Amyloid	247	247
	7–10	Non-amyloid	123	571
		Amyloid	65	228
	11–15	Non-amyloid	28	241
		Amyloid	30	245
	16–25	Non-amyloid	41	571
		Amyloid	55	778

We derived sequences of different lengths from AmyLoad database (column ‘Sequences’) and from them extracted all possible overlapping hexapeptides (column ‘Hexapeptides’). Training data sets are partially overlapping (e.g. the set 6–10 contains also sequences from the set 6). Test data sets are always non-overlapping.

### Encoding of amino acids

As previously stated, the amyloidogenicity of a given peptide may not depend on the exact sequence of amino acids but on their more general properties. To verify this hypothesis, we chose 20 different measures from the AAIndex data base^18^ describing features important in amyloidogenicity, such as size of residues, hydrophobicity, solvent surface area, frequency in *β*-sheets, and contactivity. We preferred more accurate measures introduced after 1980. The set of 20 selected physicochemical properties was supplemented by six measures of amino acid contact site propensities^19^. This gave us 26 features. Since highly correlated measures would create very similar amino acid encodings, we further reduced the number of properties to 17 by selecting measures with the absolute value of Pearson’s correlation coefficient smaller than 0.95 (see Supplemental materials, S1).

**Figure 1:**
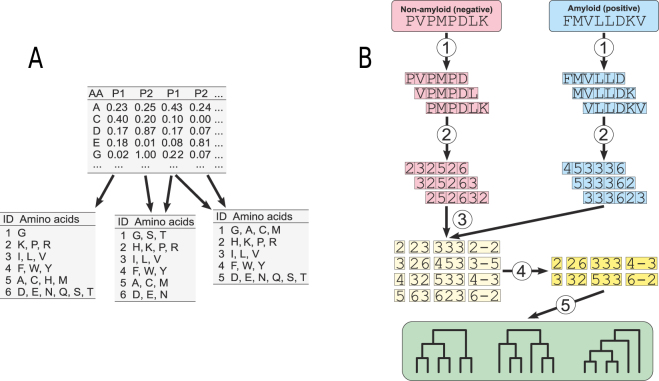
The scheme of reduced alphabets generation and n-gram extraction from studied peptide sequences. (**A**) Generation of 18,535 unique amino acid encodings using all possible combinations of selected 17 physicochemical properties. Amino acids (AA) are clustered into groups (ID) using a combination of various physicochemical properties (P1, P2, P3, P4, …). (**B**) Extraction of n-grams. (1) Extraction of overlapping hexapeptides from peptides with known amyloidicity status. (2) Encoding amino acids of hexapeptides into corresponding groups (reduced alphabet) using alphabets generated (shown in (**A**)). (3) Extraction of encoded n-grams of different types: continuous with the length from 1 to 3 residues; gapped 2-grams with a gap of the length from 1 to 3 residues; gapped 3-grams with a single gap between residues (not all possibilities are shown). (4) Selection of informative n-grams using Quick Permutation Test (QuiPT). (5) Cross-validation of encodings using random forest classifier, which is trained on the informative n-grams.

Based on these properties, we then created 524,284 encodings with different levels of amino acid alphabet reduction (three to six groups). Encodings were defined using Ward’s clustering^20^, which was performed on all combinations of the normalized values of 17 selected physicochemical properties (Fig. 1A).

The majority of encodings had at least one duplicate. In such a case, only a single representative was included in the cross-validation. After filtering out the duplicates, we obtained 18,535 unique amino acid encodings.

We evaluated the advantages of the proposed method for encoding amino acids by adding two standard encodings, ADEGHKNPQRST, C, FY, ILMV, W^21^ and AG, C, DEKNPQRST, FILMVWY, H^22^, to check if the process of amyloidogenicity does require groupings different from more general amino acid classifications. We also added the full (unreduced) amino acid alphabet to evaluate potential benefits of the alphabet reduction.

### Extraction of hexapeptides

Since we assume that a minimum length of subsequence responsible for amyloidogenicity is six residues, we extracted overlapping hexapeptides from all peptides. Each hexapeptide was labeled amyloid (positive, originating from an amyloid peptide) or non-amyloid (negative, originating from a non-amyloid peptide) (Fig. 1B1). These hexapeptides constituted our training data set.

Note that amyloid and non-amyloid elements of the set are not necessarily amyloidogenic or non-amyloidogenic. Hence, assuming that only a short part of the sequence in longer amyloids is responsible for amyloidogenicity, our method might result in many false positives in the training data set and in consequence yield inaccurate predictions, as described elsewhere^23^. To diminish this problem and facilitate the extraction of hot spots, we restricted the maximum length of peptides in the training data set to 15 amino acids. This procedure should eliminate the problem of false negatives and reduce the number of false positives. Moreover, we expect that this influence of false positives would be naturally eliminated or significantly reduced from the pattern finally found in further steps of our method. On the other hand, allowing this ambiguity, we do not eliminate many hexapeptides of potentially high amyloidogenicity, whose propensities have not been experimentally proven.

To further study the problem of the amyloidogenicity signal length, we created three training sets with sequences of varying lengths (Table 1). The smallest data set contained only sequences of the length 6. Assuming that the minimum length of the amyloidogenicity signal is six residues, we can expect no false positive hexapeptides in this set. We also created two training sets with progressively longer maximum sequence lengths of 6–10 residues and 6–15 residues.

### Extraction of encoded n-grams

From each hexapeptide we extracted encoded n-grams with the length of 1, 2, and 3. In the case of 2- and 3-grams, we separately analyzed continuous and gapped n-grams. For 2-grams, we considered n-grams with gap length from 1 to 3, whereas 3-grams could contain a single gap between the first and the second or the second and the third position (Fig. 1B3). The total number of n-grams depends on the length of the encoding and is equal to 120, 260, 480 and 798 for encodings of length 3, 4, 5, and 6, respectively. Next, the counts of n-grams were binarized (1 if n-gram was present, 0 if absent).

### Cross-validation of encodings

The encoding yielding classifier with the best ability to correctly predict amyloidogenicity of peptides was chosen during the five-fold cross-validation. We used random forests as a method for classification and trained them on the binary n-gram data drawn from the overlapping hexapeptides, considering only n-grams selected by Quick Permutation Test (QuiPT) (Fig. 1B5) described in Supplemental materials (S2). We grew the forest keeping the default number of trees (500) and the default number of variables to possibly split in each node (the rounded down square root of the total number of variables). To speed up the computation, we used the fastest implementation of random forest in **R**, the ranger package^24^.

A random forest separately considered all hexapeptides coming from a single peptide. If at least one hexapeptide extracted from a peptide was assessed as amyloidogenic, the whole sequence was denoted as amyloid. Otherwise, the peptide was classified as non-amyloid. Further, results were compared with labeled peptides to compute the performance measures.

A random assignment of peptides to subsamples in a cross-validation may result in differing numbers of hexapeptides in the subsamples, because longer peptides yield more hexapeptides than shorter ones. Therefore, we repeated the cross-validation fifteen times for each classifier to obtain more precise estimates of performance measures. We considered three length ranges of sequences in the training sets, 6, 6–10 and 6–15 residues, to evaluate if our classifiers are able to use decision rules extracted from sequences of different lengths to correctly classify longer or shorter sequences. During the cross-validation, for each training set (6, 6–10 and 6–15) we randomly assigned peptides to 5 subsamples. Using each subsample we tested the classifier trained on other subsamples. Additionally, classifiers obtained in cross-validation were also tested on the data set of long peptides (16–25), also splitted randomly into 5 subsamples. Later, we computed performance measures for peptides in the test subsample separately for each length group (6, 7–10, 11–15, 16–25).

To choose the most adequate amino acid encoding, we ranked the values of the Area Under the receiver operating characteristic Curve (AUC) for each particular classifier, assigning the rank 1 for the best AUC, rank 2 for the second best AUC, and so on, and various ranges of the sequence length in the test data set. The encoding with the lowest sum of ranks from all sequence length categories was selected as the best. For this encoding, we chose the range of peptide lengths in the training set that provided the best AUC in the cross-validation.

### Benchmark of AmyloGram

The best-performing encoding chosen during the cross-validation of encodings was used to train AmyloGram, the n-gram based predictor of peptide amyloidogenicity. To compare the performance of AmyloGram and other predictors of amyloids, we used the external data set *pep424* ^9^. We did not filter peptides using pairwise identity, because this criterion does not reflect likelihood of undergoing amyloid aggregation. Peptides common to both *pep424* and AmyLoad were removed from the training data set, leaving 222 positive sequences and 739 negative sequences in the training data set. No other redundancy level can be assumed in these short fragments since in many cases the difference of one residue is enough to discriminate between amyloid and non-amyloid peptides (see Supplemental materials, S5). The sequences in this set were all longer than 5 and shorter than 15 residues. Aside from the removal of sequences, the AmyloGram training set was identical to the training of classifiers during the cross-validation. The parameters of QuiPT and random forest algorithms were kept the same.

We removed peptides shorter than six amino acids from the *pep424* data set as our model of amyloidogenicity assumes the minimum length of six residues. Such a change should not affect the outcome of the comparison because only about 1% (5 sequences) were removed. To separately assess the benefit of using the n-gram analysis and the full, unreduced, 20 amino acid alphabet, we also benchmarked predictors trained on n-grams extracted from each of the three training sequence length ranges.

## Results and Discussion

### Performance of the best encoding

**Figure 2:**
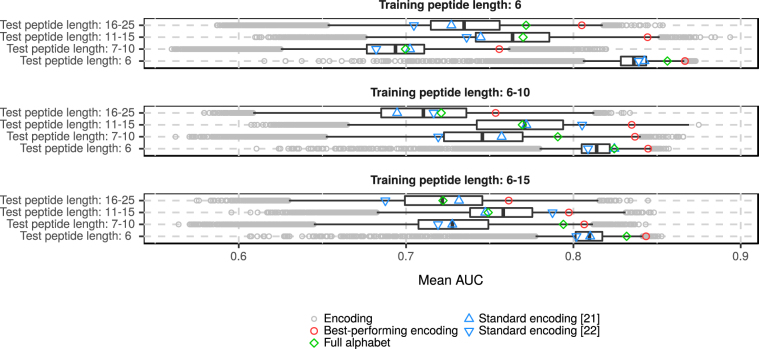
Distribution of mean AUC values of classifiers with various encodings for every possible combination of training and testing data set including different lengths of sequences. The left and right ends of boxes correspond to the 0.25 and 0.75 quartiles. The bar inside the box represents the median. The gray circles correspond to the encodings with the AUC outside the 0.95 confidence interval.

The AUC of the predictor based on the best-performing encoding was always in the fourth quartile of all AUC values (Fig. 2). It had the highest AUC (0.8667) in classification of the shortest sequences (with a length of 6 residues) when the training set consisted of sequences of the same length. This result occurs most probably from homogeneity of the short peptide set.

The most problematic result was the correct prediction of the amyloidogenicity in the longest peptides, ranging from 16 to 25 residues, when the algorithm was trained on longer peptides, i.e. the 6–10 and 6–15 data sets. Here the AUC value did not exceed 0.77. This weak performance results from more complex organization of longer amylogenic peptides. In such peptides, only a very specific region of residues might be responsible for the creation of harmful aggregates. In this case, when overlapping hexapeptides are extracted, only part of them may carry the true signal of amyloidogenicity but all of them are marked as amyloids.

In addition to the high AUC, the best encoding also had very good sensitivity and specificity, regardless of the sequence lengths in the training and tested sets (see Supplemental materials, S3). Classifiers trained on peptides of length 6 tended to have the best specificity, whereas predictors trained on longer sequences had the best sensitivity. Although the classifiers trained on the six-residue long sequences generally had a better AUC, their training on the sequences from six to ten residues seemed to yield the most balanced classifiers with the optimal sensitivity and specificity.

We also evaluated classifiers based on the full, unreduced, amino acid alphabet. In most cases, the AUCs of these classifiers were in the fourth quartile of the AUC values (Fig. 2). Nevertheless, they never predicted amyloidogenicity better than the best classifier based on the reduced alphabet. This implies that the amyloidogenicity can be described more accurately using less than the full set of 20 amino acids.

Similar to the best-performing encoding, the sensitivity of classifiers based on the full amino acid alphabet decreased with the length of sequences in the training data set (see Supplemental materials, S3). Furthermore, these classifiers always had the worst sensitivities among all analyzed predictors, especially when tested on the longer amyloids. This means that the full amino acid alphabet recognized non-amyloidogenic sequences easier than amyloidogenic sequences.

Standard encodings included in the cross-validation often have AUC values below the median. This implies that although the amyloidogenicity can be described by a reduced amino acid alphabet, such an alphabet must consider only very special physicochemical properties of residues and cannot be too general.

### The best-performing encoding and important n-grams

In total, eleven combinations of physicochemical properties created the best performing encoding. Only four features appeared in all combinations: hydrophobicity index^25^, average flexibility indices (a normalized fluctuational displacement of an amino acid residue)^26^, polarizability parameter^27^ and thermodynamic *β*-sheet propensity^28^.

The best encoding chosen in the analysis consists of six amino acid subgroups, each characterized by distinct and specific properties (Table 2). Subgroup III contains strongly hydrophobic amino acids. Amino acids from subgroup IV also have aromatic properties. On the other hand, the most hydrophilic amino acids are in subgroups II and VI. The former includes two strongly basic amino acids, whereas the latter has two acidic and four polar residues. Subgroup I includes only glycine, which is the smallest amino acid and the most flexible. By average, relatively flexible amino acids are also present in subgroup II, whereas the least flexible amino acids are in subgroups IV and V. Glycine has the lowest propensity to form *β*-sheets whereas subgroups III and IV the highest.

**Table 2:** The best-performing encoding.

Subgroup ID	Amino acids
I	G
II	K, P, R
III	I, L, V
IV	F, W, Y
V	A, C, H, M
VI	D, E, N, Q, S, T

**Figure 3:**
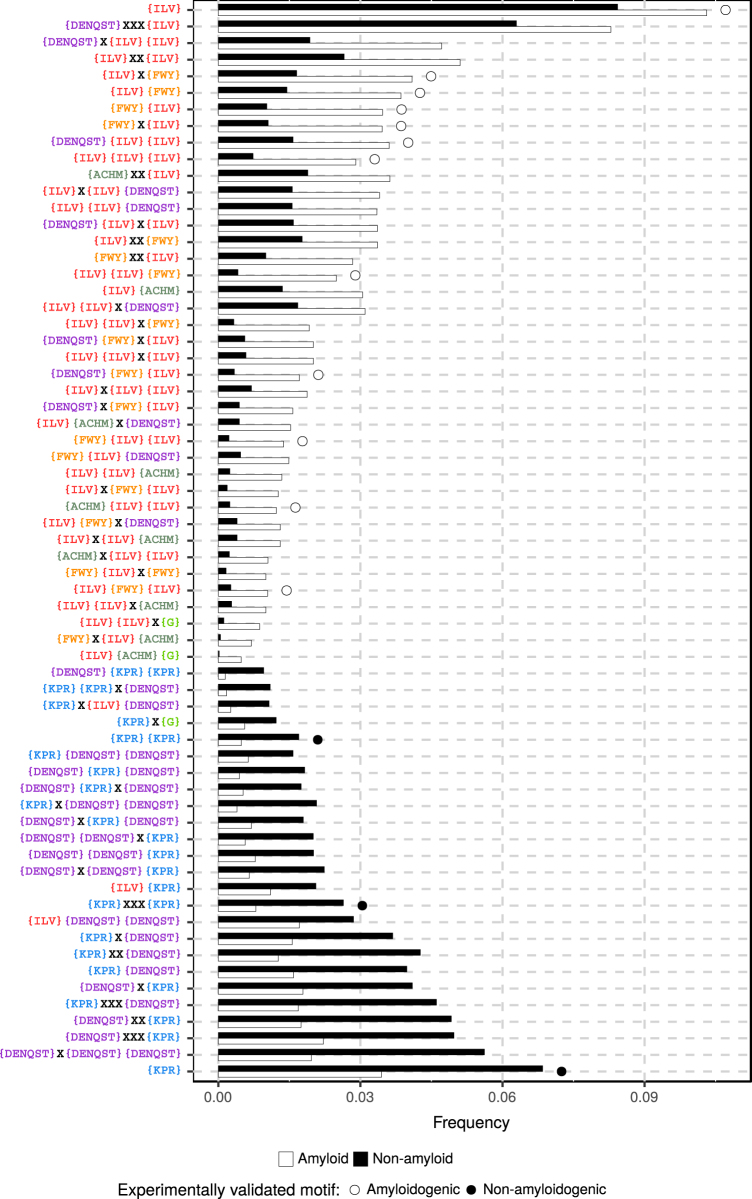
The frequency of important n-grams used by the best-performing classifier in amyloid and non-amyloid sequences. Amino acids possible on a given position of the n-grams are specified inside the brackets. X denotes any amino acid. The frequency was computed using the total number of occurrences divided by the number of possible n-grams of their length. Open and closed circles denote experimentally validated n-grams occurring in motifs found in amyloidogenic and non-amyloidogenic sequences, respectively^30^.

We found 65 n-grams with p-values smaller than 0.05 in the QuiPT test in all repetitions of cross-validation, regardless of the lengths of sequences in the training set (Fig. 3). The frequency of the n-grams was computed for all sequences derived from AmyLoad. The n-grams typical of amyloidogenic sequences (with the highest frequency occurrence in amyloids) include mostly highly hydrophobic amino acids with tendency to form *β*-structures, from subgroups III and IV. The n-grams occurring frequently in amyloids have often repeats of amino acids from subgroup III, suggesting that the presence of these amino acids in the vicinity might be one of the most effective predictors of amyloidogenicity. This result confirms experimental findings of other groups^29^.

In contrast, n-grams typical of non-amyloidogenic peptides have mostly amino acids belonging to subgroups II and VI. These subgroups include strongly hydrophilic and highly flexible amino acids (K, P, R, D, E), which hamper the formation of *β*-structures.

Of the 65 most informative n-grams, 15 (23%) were also found in the motifs validated experimentally for amyloidogenic and non-amyloidogenic peptides^30^. The peptides used in this study are included in the AmyLoad database, thus n-gram analysis is at least partially able to find the patterns in validated sequences.

**Figure 4:**
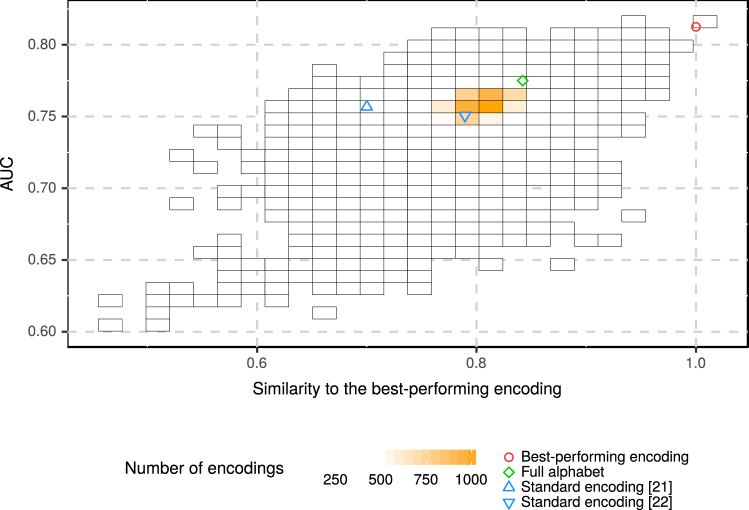
Similarity and AUC of the reduced alphabets studied in the cross-validation. Classifiers the most similar to the best-performing classifier have the highest values of AUC. The color of the square is proportional to the number of alphabets in its area.

To compare the best-performing encoding to other encodings, we computed the similarity between them (Fig. 4) using the measure introduced specifically for reduced amino acid alphabets^31^. The value of AUC is significantly lower for more distant encodings (0.5096 Pearson’s correlation coefficient, p-value < 2.2×10^−16^). Such relationship indicates that the best-performing encoding was not found by chance and inclusion of properties reflected by this encoding improves the prediction of amyloids.

### Benchmark of AmyloGram

Benchmarking included AmyloGram as well as three peer-reviewed predictors of amyloidogenicity, the physical models PASTA 2.0^9^, FoldAmyloid^5^, and the neural network based APPNN^32^. None of these methods use a reduced amino acid alphabet, but APPNN codes amino acids using the exact values of their physicochemical properties. Some other known classifiers were not included in the benchmark because their performance on the *pep424* data set is already known and inferior to the performance of PASTA 2.0 and FoldAmyloid^9^. narrow

We analyzed AUC, Matthew’s Correlation Coefficient (MCC), sensitivity and specificity (Table 3). We used default settings for FoldAmyloid and APPNN. PASTA 2.0 evaluated the input data in the ‘Peptides’ mode, which is advised by its authors for peptide.

**Table 3:** Results of benchmark on the pep424 data set for PASTA 2.0, FoldAmyloid, APPNN, and AmyloGram trained on n-grams extracted for the full amino acid alphabet and for sequences with the length specified in the brackets.

Classifier	AUC	MCC	Sensitivity	Specificity
AmyloGram (6)	0.8856	0.6057	0.6779	0.9037
full alphabet (6)	0.8411	0.5427	0.4966	**0**.**9593**
AmyloGram (6–10)	**0**.**8972**	**0**.**6307**	0.8658	0.7889
full alphabet (6–10)	0.8581	0.5698	0.7517	0.8259
AmyloGram (6–15)	0.8728	0.5420	**0**.**9463**	0.6111
full alphabet (6–15)	0.8610	0.5490	0.8188	0.7519
PASTA 2.0	0.8550	0.4291	0.3826	0.9519
FoldAmyloid	0.7351	0.4526	0.7517	0.7185
APPNN	0.8343	0.5823	0.8859	0.7222

Since PASTA 2.0 does not return a probability of belonging to a specific category, we normalized the output data to compute the AUC values. The advised energy threshold (−5) was normalized in the same manner and used as cut-off in computations of specificity, sensitivity and MCC. The resulting value of specificity 0.9519 is close to the value provided by its authors (0.95) and assures correctness of our computations. For other classifiers, including AmyloGram, we assumed a default 0.5 cut-off.

For the studied data set, the n-gram extraction method is efficient enough to produce classifiers that outperform other published methods. AmyloGram showed the highest AUC and MCC among all tested classifiers. Note that it outperformed its counterparts trained on the full amino acid alphabet and that it is the most balanced tool among all analyzed classifiers, having the best specificity/sensitivity trade-off, as indicated by the value of MCC.

The specificity of AmyloGram is lower than the specificity of PASTA 2.0 when the threshold value of PASTA 2.0 is optimized for 0.95 specificity. If we assume for AmyloGram the same threshold for the specificity, our classifier has a higher sensitivity (0.5518) than PASTA 2.0. Therefore, if we assume such thresholds to both predictors, they will detect true non-amyloids with the same specificity but AmyloGram will predict more true amyloids.

Two of the three AmyloGram classifiers trained on full alphabet n-grams had AUCs higher than PASTA2 and all three were more successful than either FoldAmyloid or APPNN. They also maintained the high specificity observed previously during cross-validation. Further, the AmyloGram classifier based on the reduced amino acid alphabet always outperformed that based on the full alphabet.

Among all considered predictors of amyloidogenicity, APPNN had the highest sensitivity. Nevertheless, its AUC was lower than the AUCs of all the n-gram-based predictors, as well as that of PASTA2, indicating lower overall performance.

AmyloGram is trained to predict amyloidogenic, not amyloidic regions. Hence, we did not test it on the *reg33* data set, which is commonly used to evaluate the amyloid propensity of the full peptide^33^.

## Conclusions

The description of peptides by short sub-sequences (n-grams) followed by the reduction of the amino acid alphabet allowed us to create the efficient predictor of amyloidogenic sequences, named AmyloGram. One of the strengths of this approach is its highly interpretable outcome, because our methods provide explicitly short motifs relevant to amyloidogenicity of peptides and discriminating amyloids from non-amyloids. Sixty-five important n-grams revealed that mostly aliphatic and nonpolar amino acids (isoleucine, leucine and valine), together with aromatic and also hydrophobic amino acids (phenylalanine, tyrosine, tryptophan) are good predictors of amyloid peptides. Polar and hydrophilic residues (K, P, R) never occur in n-grams associated with amyloidogenicity which is confirmed by experimental studies. On the other hand, polar residues such as D, E, N, Q, S, and T are present both in amyloidogenic and non-amyloidogenic sequences. It seems plausible, that the latter amino acids are necessary for the proper formation of some hot spots, but must be complemented by hydrophobic and aromatic residues. That means that hot spots are not completely hydrophobic and may contain a fraction of hydrophilic residues with the exclusion of known breakers of *β*-structures such as lysine, proline and arginine.

Our studies confirm that the most important physicochemical properties associated with amyloidogenicity are hydrophobicity and tendency to form *β*-sheets. We additionally discovered that amino acid flexibility can also sufficiently discriminate amyloid and non-amyloid peptides. The aggregating peptides tend to have more amino acid residues with lower flexibility which seems to be confirmed by experimental studies^34^. However, recent findings indicate that amyloidic core may be flexible enough to form ring-like structures^35^. In this light, it could be considered that the result indicating lower flexibility of amyloids could also stem from the bulkiness of amino acids constituting their sequences, since bulkiness and flexibility measures are correlated (Supplement, Table S7 and Figs S3–S5). The n-gram analysis also showed sequential patterns of the amino acid groups appearing in the amyloids. Among the most informative n-grams we identified 15 that were independently confirmed experimentally.

It should be noted that prions are very special type of amyloid proteins for which somehow different physicochemical rules probably hold^36^. This is why methods developed for general amyloid datasets do not work well with prions. Since our method was trained on a very general dataset of amyloids, in which prions constitute a very tiny part, it is not intended for prions.

Our findings are helpful in understanding the process of amyloid aggregation and recognition of peptides susceptible to the formation of amyloid aggregates involved in various diseases. Moreover, they might be employed in the creation of synthetic amyloid peptides. We anticipate that the n-gram analysis we have described is versatile enough to be applied in other areas of protein function prediction.

## Electronic supplementary material

Supplemental data
