# Supplementary material for: Amyloidogenic motifs revealed by n-gram analysis
Source: Sci Rep. 2017 Oct 11;7:12961. doi: 10.1038/s41598-017-13210-9 (PMC5636826; doi:10.1038/s41598-017-13210-9)
Supplement: Supplementary file 1 — Supplemental data [file 41598_2017_13210_MOESM1_ESM.pdf]

Amyloidogenic motifs revealed by n-gram analysis

Supplemental materials

Michał Burdukiewicz, Piotr Sobczyk, Stefan Rödiger, Anna Duda-Madej,
Paweł Mackiewicz and Małgorzata Kotulska

Contents

S1 Physicochemical properties 2

S2 Quick Permutation Test (QuiPT) 3

S3 Sensitivity and specificity 4

S3.1 Training peptide length: 6, test peptide length: 6 5
S3.2 Training peptide length: 6, test peptide length: 7-10 6
S3.3 Training peptide length: 6, test peptide length: 11-15 7
S3.4 Training peptide length: 6, test peptide length: 16-25 8
S3.5 Training peptide length: 6-10, test peptide length: 6 9
S3.6 Training peptide length: 6-10, test peptide length: 7-10 10
S3.7 Training peptide length: 6-10, test peptide length: 11-15 11
S3.8 Training peptide length: 6-10, test peptide length: 16-25 12
S3.9 Training peptide length: 6-15, test peptide length: 6 13
S3.10Training peptide length: 6-15, test peptide length: 7-10 14
S3.11Training peptide length: 6-15, test peptide length: 11-15 15
S3.12Training peptide length: 6-15, test peptide length: 16-25 16

S4 Bootstrap confidence intervals for benchmark 17

S5 Pairwise sequence identity between training and benchmark data sets 20

S6 Jackknife test 22

S7 Amino acid flexibility/rigidity and size 22

S8 Bibliography 22

S1 Physicochemical properties

Table 1: Selected 17 physicochemical properties used to create amino acid encodings.

Category	Property
Contactivity	Average flexibility indices (Bhaskaran and Ponnuswamy, 1988)
Contactivity	14 Å contact number (Nishikawa and Ooi, 1986)
Contactivity	Accessible surface area (Radzicka and Wolfenden, 1988)
Contactivity	Buriability (Zhou and Zhou, 2004)
Contactivity	Contact frequency in proteins from class β, cutoff 12 Å, separation 5 Å (Wozniak and Kotulska, 2014)
Contactivity	Contact frequency in proteins from class β, cutoff 12 Å, separation 15 Å (Wozniak and Kotulska, 2014)
β-frequency	Average relative probability of inner beta-sheet (Kanehisa and Tsong, 1980)
β-frequency	Relative frequency in β-sheet (Prabhakaran, 1990)
β-frequency	Thermodynamic β-sheet propensity (Kim and Berg, 1993)
Hydrophobicity	Hydrophobicity index (Argos *et al.*, 1982)
Hydrophobicity	Optimal matching hydrophobicity (Sweet and Eisenberg, 1983)
Hydrophobicity	Hydrophobicity-related index (Kidera *et al.*, 1985)
Hydrophobicity	Scaled side chain hydrophobicity values (Black and Mould, 1991)
Polarity	Polarizability parameter (Charton and Charton, 1982)
Polarity	Mean polarity (Radzicka and Wolfenden, 1988)
Size	Average volumes of residues (Pontius *et al.*, 1996)
Stability	Side-chain contribution to protein stability (kJ/mol) (Takano and Yutani, 2001)

S2 Quick Permutation Test (QuiPT)

Permutation tests are commonly used for filtering important n-grams testing, the hypothesis that an occurrence of n-gram and a value of a target are independent. However, exhaustive testing of permutations is computationally expensive and, as a result, they often become one of the most limiting factors in these kinds of analyses. Therefore, we developed the Quick Permutation Test which effectively filters n-gram features, without requiring exhaustive testing, and using the information gain (mutual information) as the criterion of the importance of a specific n-gram. Using QuiPT we selected the most discriminating n-grams extracted from the hexapeptides of the training data set. Again, the counts of n-grams were binarized (1 if n-gram was present, 0 if absent). Only n-grams with p-value smaller than 0.05 were assumed to be informative.

Consider a contingency table for a target y and a feature x (Tab. 2). For example, the entry $n_{1,0}$ is the number of cases when the target is 1 and the feature is 0.

Table 2: A contingency table for a target y and a feature x.

target / feature	1	0	total
1	$n_{1,1}$	$n_{1,0}$	$n_{1,\cdot}$
0	$n_{0,1}$	$n_{0,0}$	$n_{0,\cdot}$
total	$n_{\cdot,1}$	$n_{\cdot,0}$	n

Under the hypothesis that x and y are independent, the probability of observing such a contingency table is given by the multinomial distribution in which all probabilities depend only on marginal distributions. The idea of the permutation test is to reshuffle labels of features and targets, while keeping the fixed total number of positives for features and targets. When we impose this constraint on the multinomial distribution, then the probability of occurrence for a given contingency table depends on only one entry, $n_{1,1}$, which is fairly easy to compute. After computing Information Gain (IG) for each possible value of $n_{1,1} \in [0, \min(n_{\cdot,1}; n_{1,\cdot})]$, we get the distribution of Information Gain under the hypothesis that the target and feature are independent. We reject the null hypothesis of independence, if the IG for the tested feature is above the required quantile from the IG distribution.

The analytic formula for the distribution enables to perform the permutation test much quicker. Furthermore, we get exact quantiles even for extreme tails of the distribution, which is not guaranteed by random permutations. For example, for the test at the level $\alpha = 10^{-8}$, which can often occur in the corrections for multiple testing, the standard deviation of quantile estimate in the permutation test, $\frac{p(1-p)}{m}$, is roughly equal to α itself even for a very large number of permutations like $m = 10^8$.

In the context of n-gram data, we can further speed up our algorithm. Note that test statistics depends only on $n_{\cdot,1}$, i.e., the number of positive cases in the feature when the target y is common for testing all n-gram features. Although we test millions of features, there are only a few distributions that we need to compute because the usual number of positives in n-gram feature is small. We take advantage of this fact and compute quantiles only for this small number of distributions. Therefore, the complexity of our algorithm is roughly equal to $O(n \cdot p)$, where n and p represents the number of features and number of positives, respectively.

Last, let us point out that QuiPT is very similar to Fisher's exact test. From the derivation provided in reference (Lehmann and Romano, 2008) and elsewhere, it becomes obvious that QuiPT is a heuristics for an unsolved problem of a two-tailed Fisher's exact test. In this heuristics, the extremity of a contingency table is defined by its information gain.

S3 Sensitivity and specificity

Sensitivity and specificity of classifiers with various encodings for every possible combination of sequence lengths in the training and testing data sets.

The classifier based on the best-performing encoding always have a good specificity and sensitivity. The color of the square is proportional to the number of encodings in its area. Points represent classifiers based on special encodings: the best-performing encoding, full amino acid alphabet and two two standard encodings, ADEGHKNPQRST, C, FY, ILMV, W (Kosiol *et al.*, 2004) and AG, C, DEKNPQRST, FILMVWY, H (Melo and Marti-Renom, 2006).

S3.1 Training peptide length: 6, test peptide length: 6

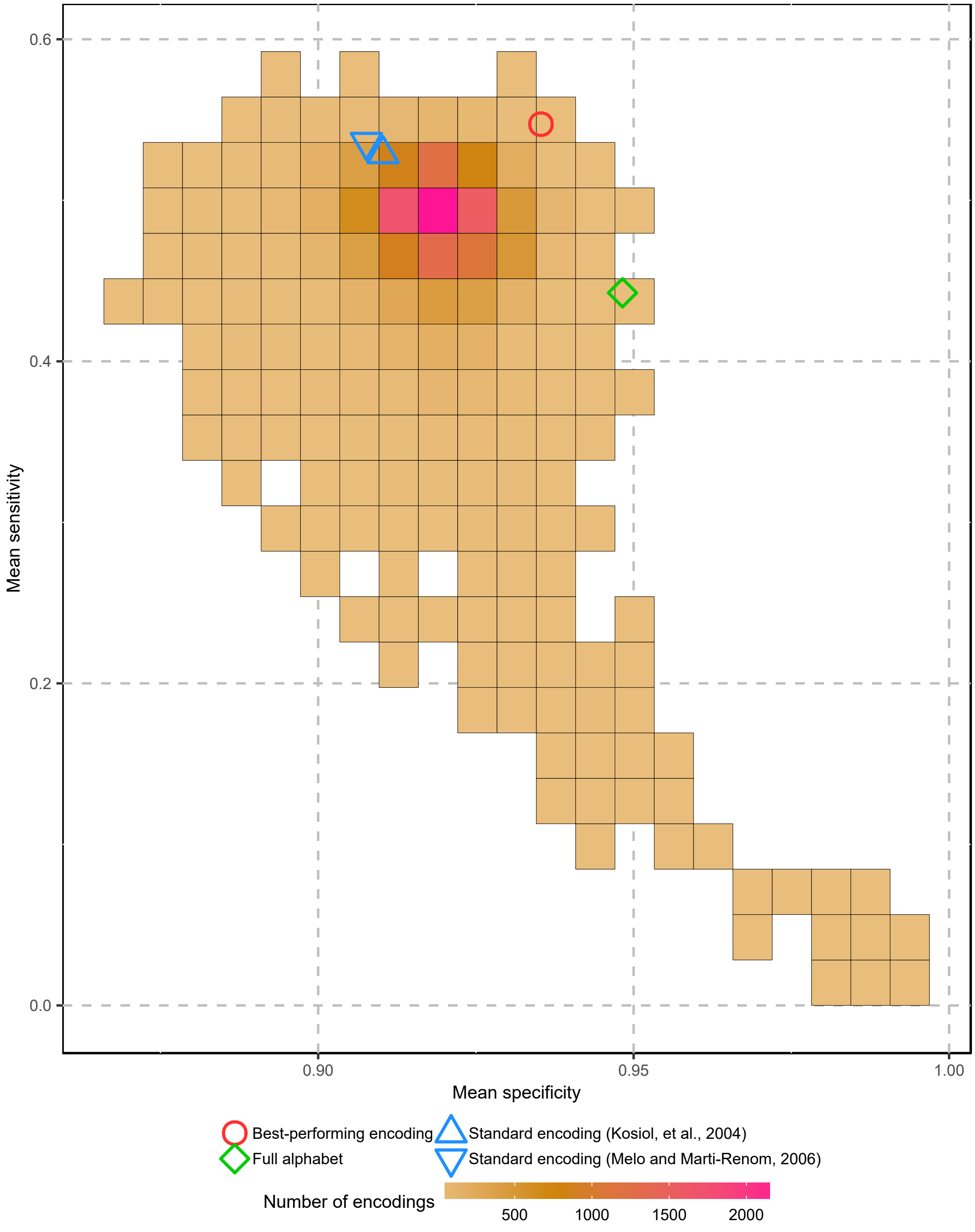

S3.2 Training peptide length: 6, test peptide length: 7-10

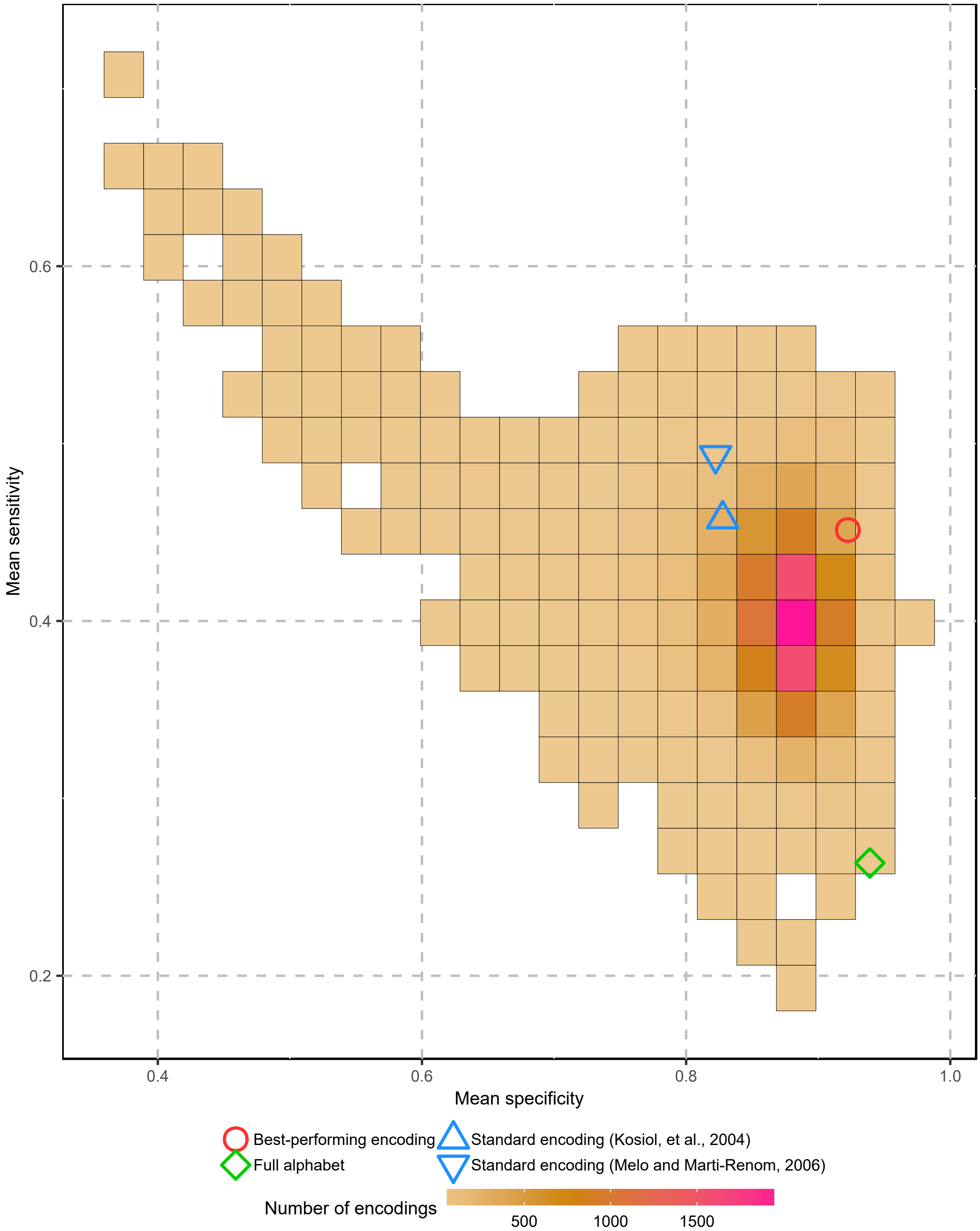

S3.3 Training peptide length: 6, test peptide length: 11-15

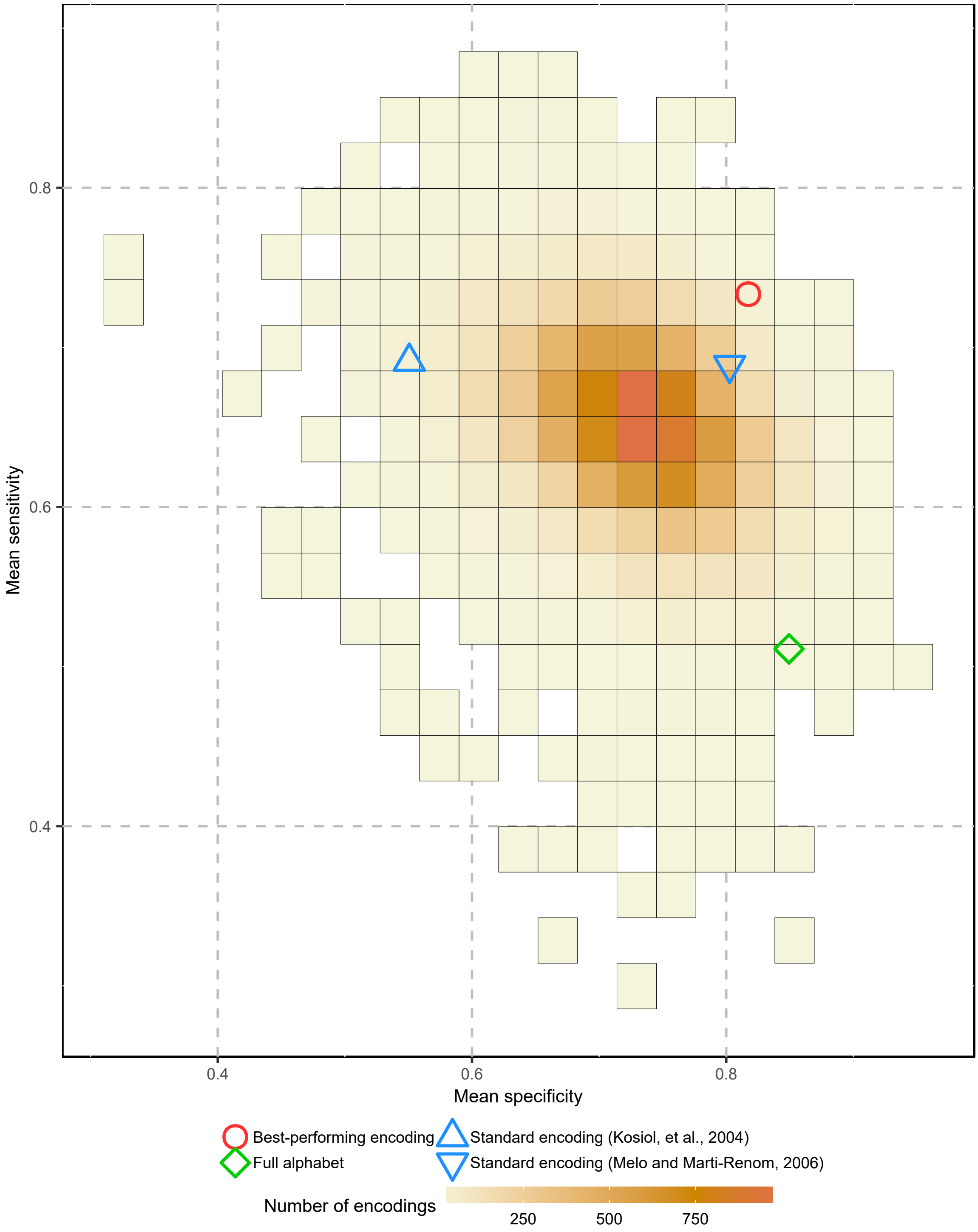

S3.4 Training peptide length: 6, test peptide length: 16-25

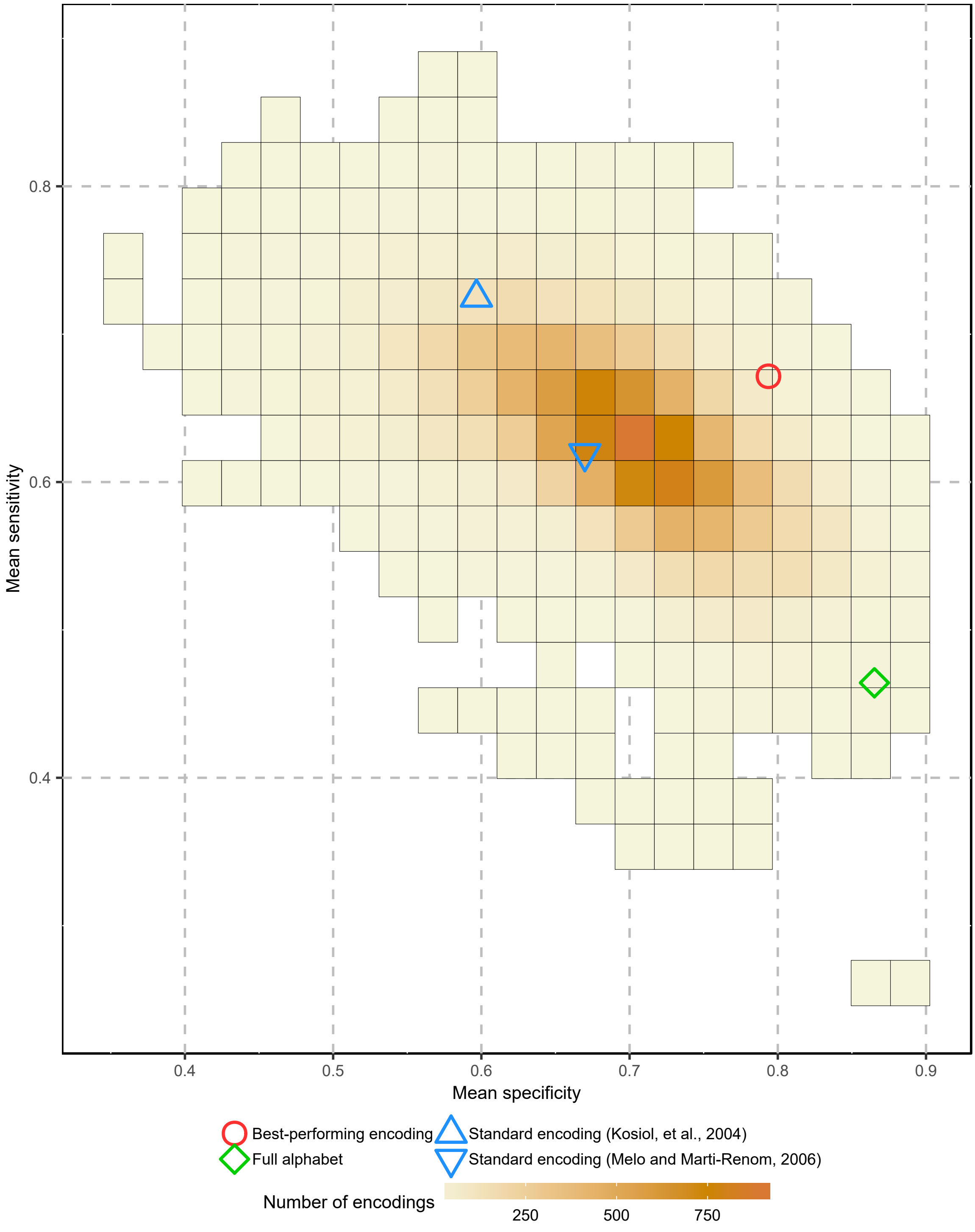

S3.5 Training peptide length: 6-10, test peptide length: 6

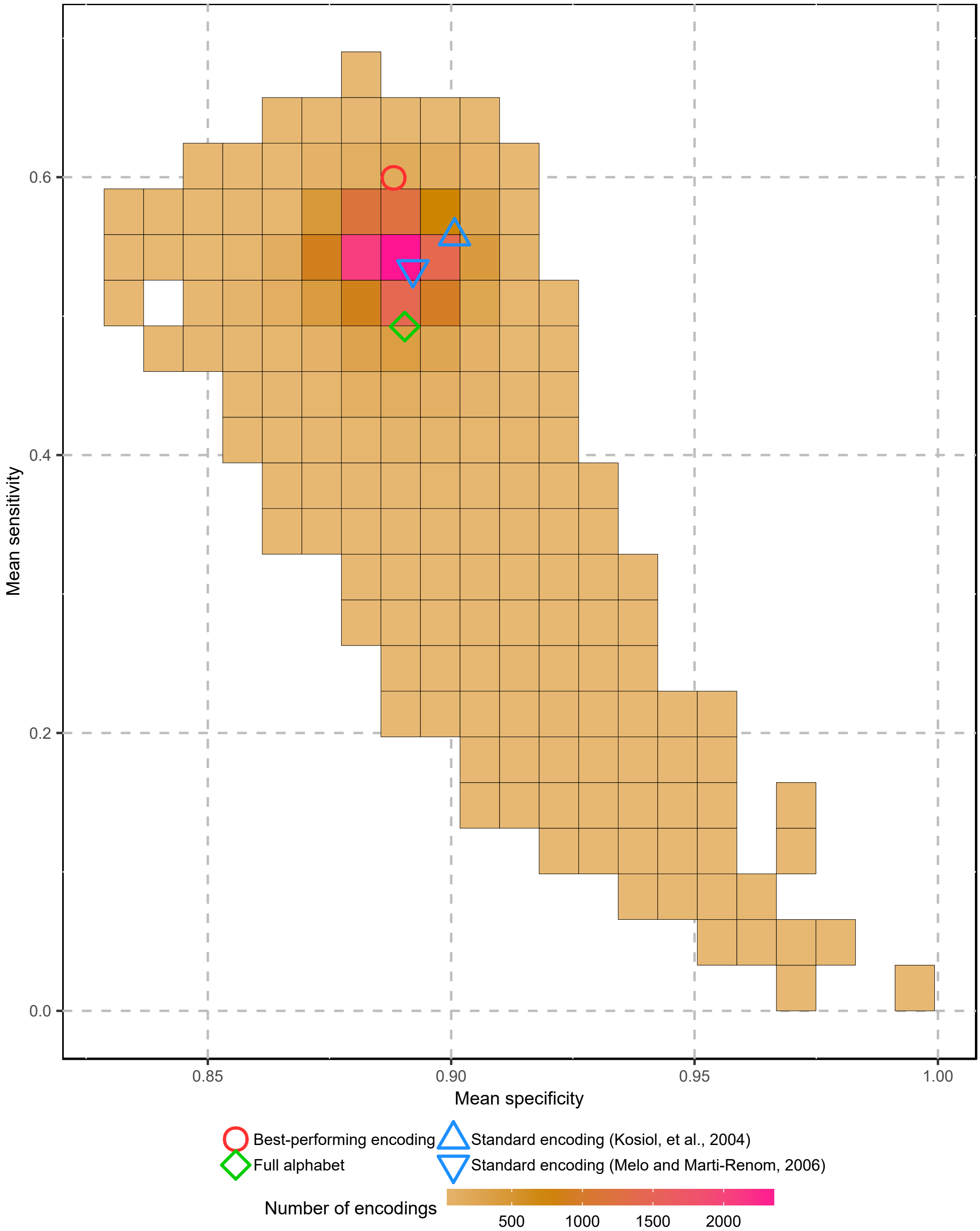

S3.6 Training peptide length: 6-10, test peptide length: 7-10

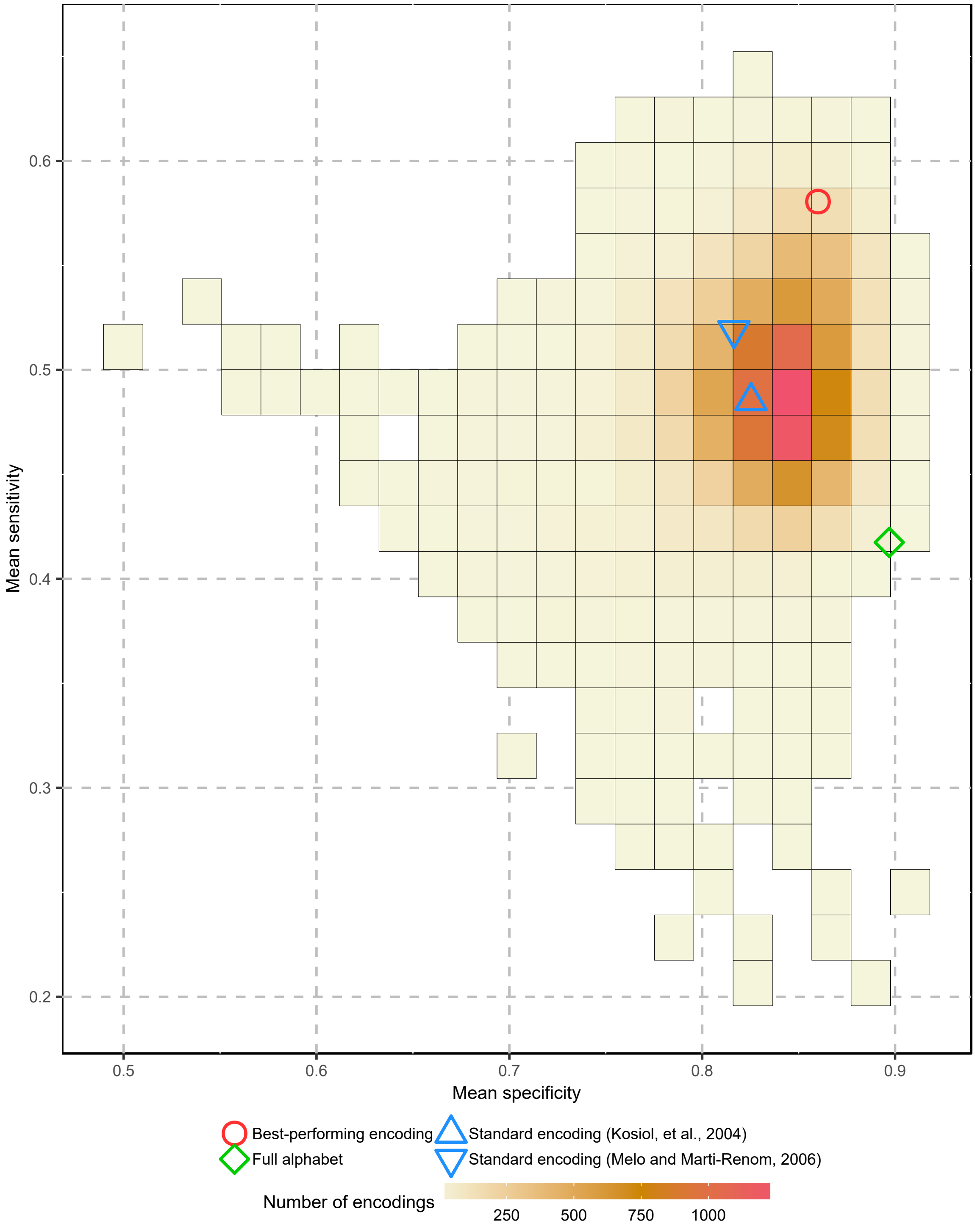

S3.7 Training peptide length: 6-10, test peptide length: 11-15

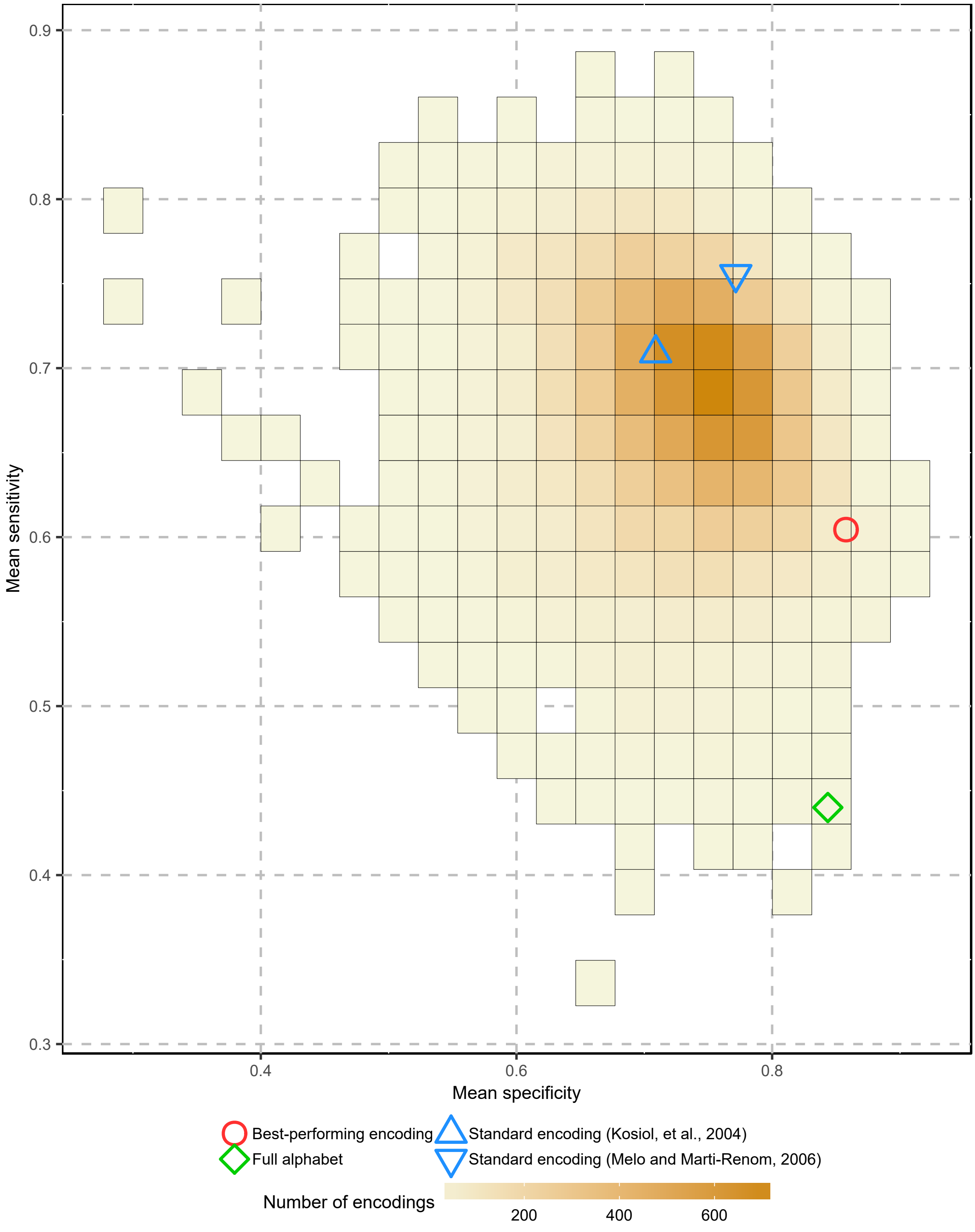

S3.8 Training peptide length: 6-10, test peptide length: 16-25

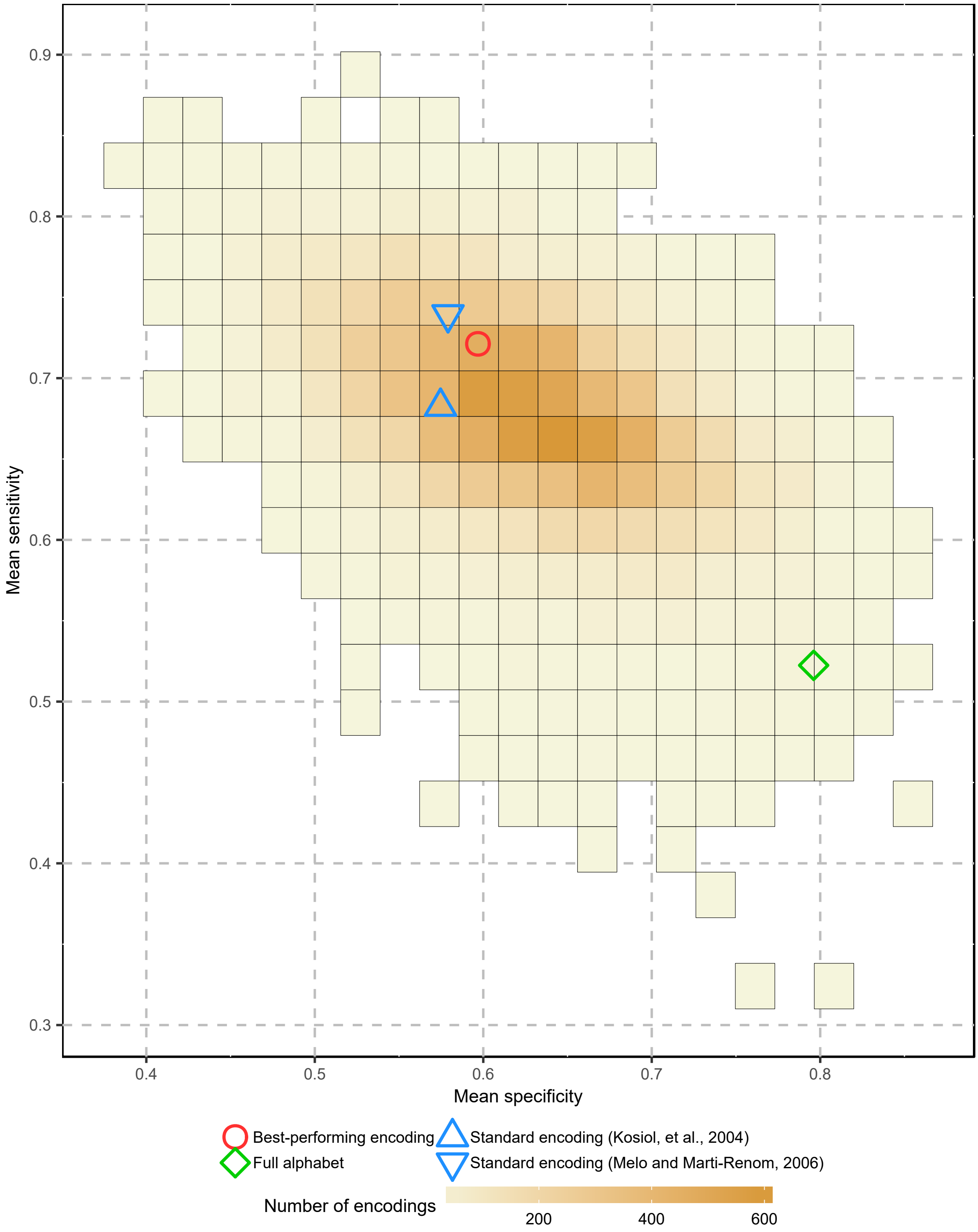

S3.9 Training peptide length: 6-15, test peptide length: 6

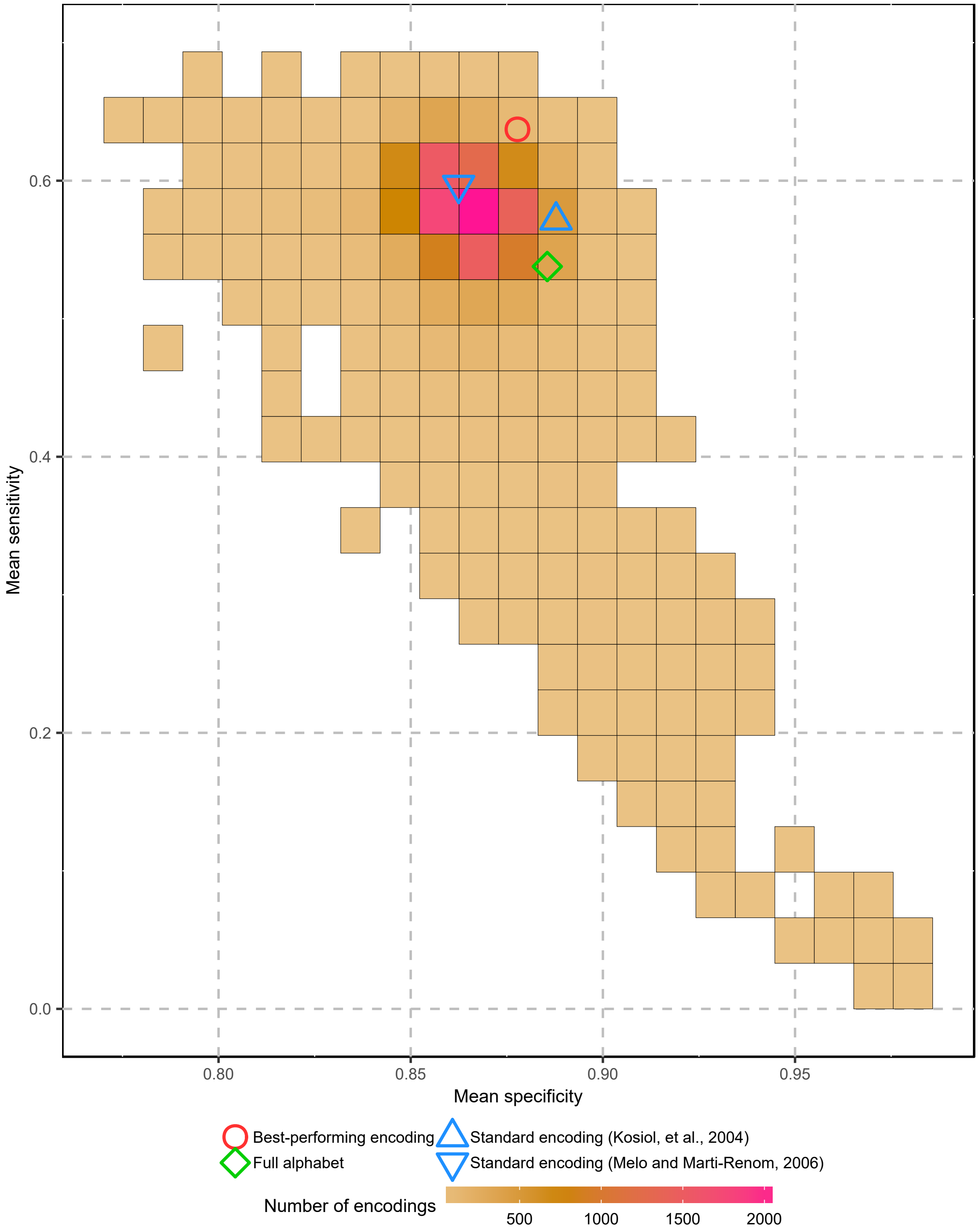

S3.10 Training peptide length: 6-15, test peptide length: 7-10

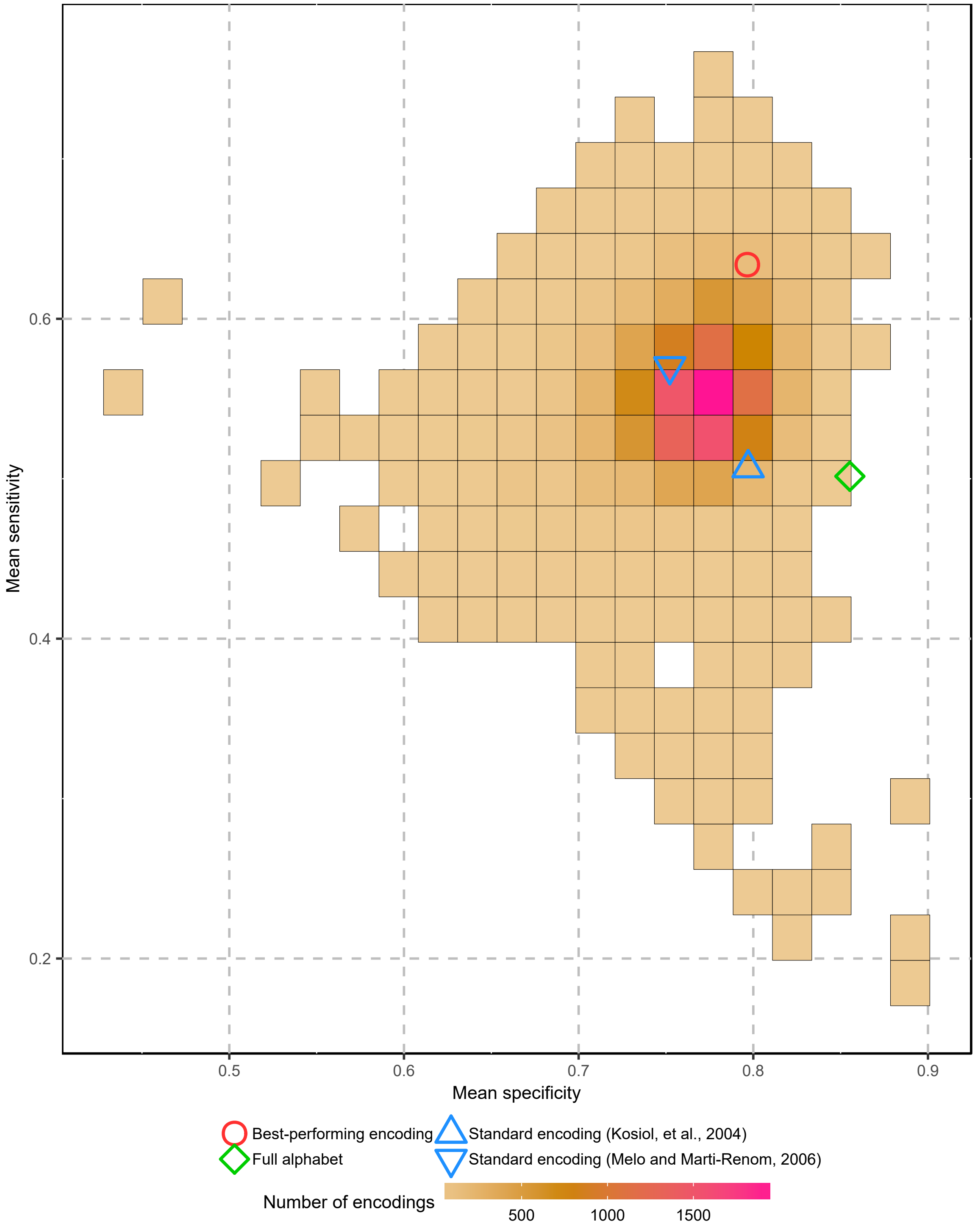

S3.11 Training peptide length: 6-15, test peptide length: 11-15

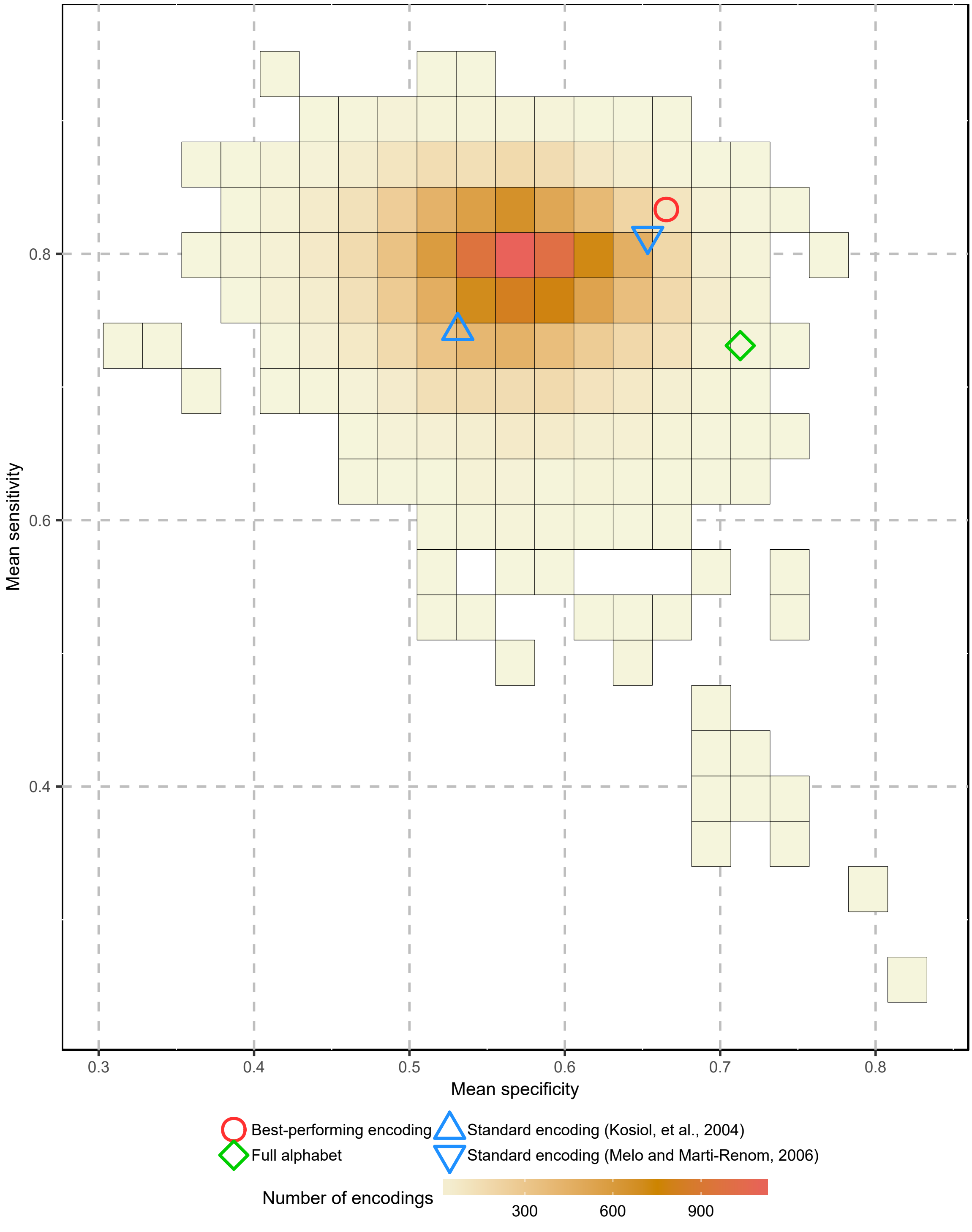

S3.12 Training peptide length: 6-15, test peptide length: 16-25

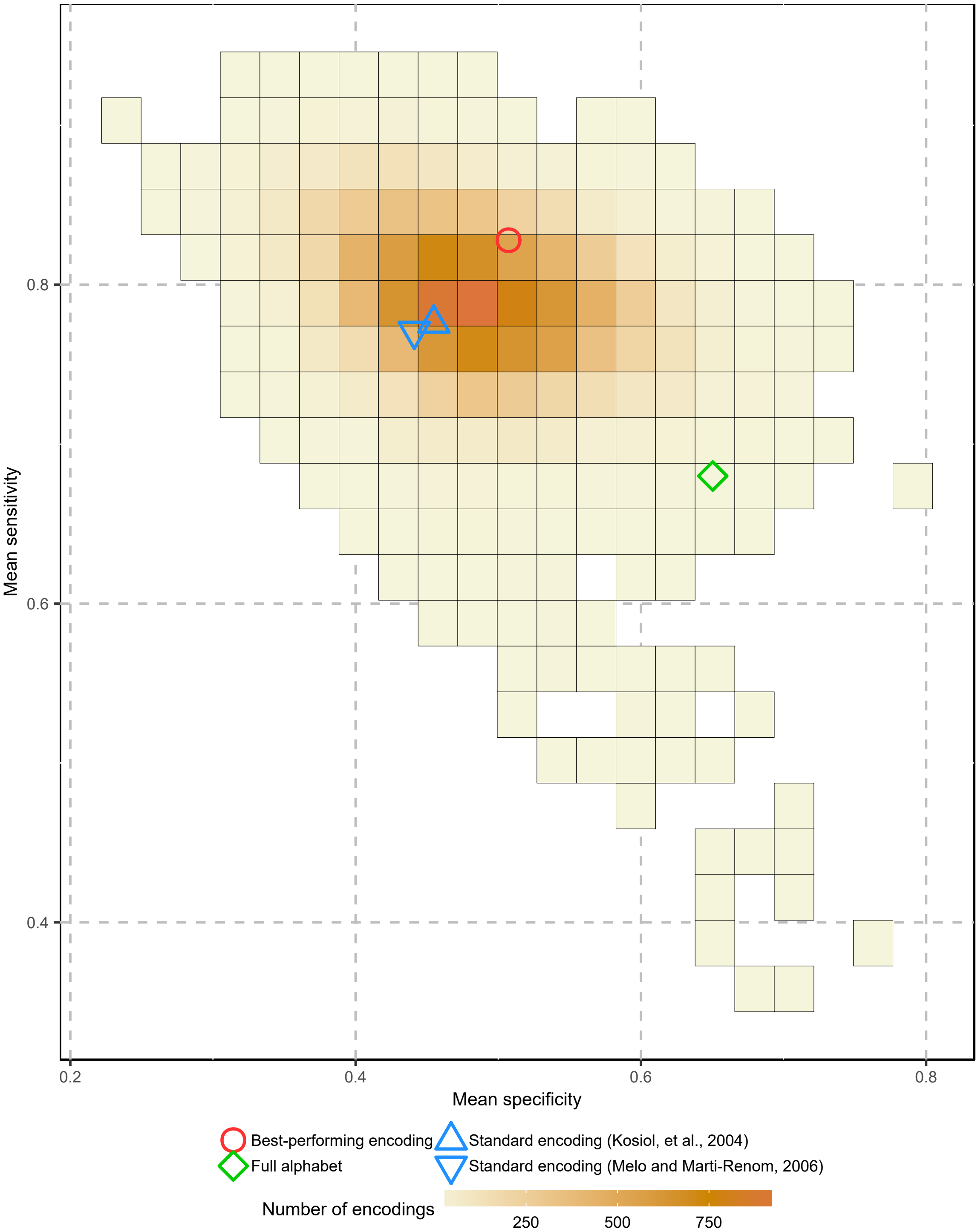

S4 Bootstrap confidence intervals for benchmark

We computed 0.95 confidence intervals for all classifiers by bootstrapping results of the benchmark(Efron and Tibshirani, 1994). Briefly, predictions returned by classifiers were sampled with replacement number of times equal to the total number of predictions. For each bootstrap sample we computed performance measures. Repeating the procedure 1000 times, we obtained a robust estimate of 95% confidence intervals adjusted for multiple comparison using Dunn – Šidák correction, therefore significantly different values of performance measures can be distinguished (see Tab. 3 and Fig. 1).

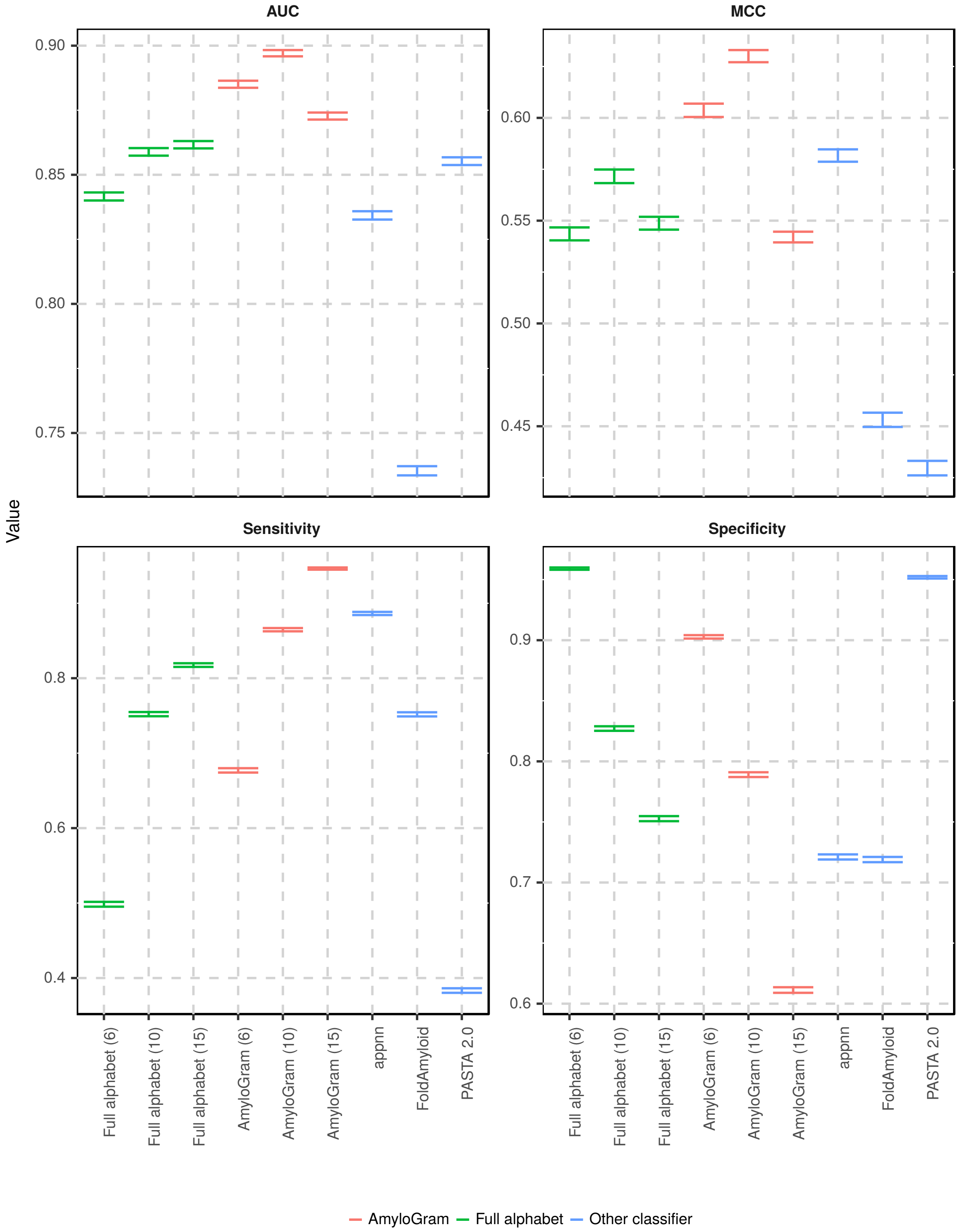

Figure 1: 0.95 bootstrap confidence intervals for benchmark results adjusted for multiple comparisons.

Table 3: The mean values of performance measures and their confidence intervals obtained in the bootstrap.

Measure	Classifier	Mean	Lower bound	Upper bound
AUC	PASTA 2.0	0.8553	0.8568	0.8538
	FoldAmyloid	0.7354	0.7371	0.7336
	appnn	0.8343	0.8359	0.8327
	AmyloGram (15)	0.8727	0.8741	0.8714
	AmyloGram (10)	0.8971	0.8983	0.8959
	AmyloGram (6)	0.8851	0.8864	0.8837
	Full alphabet (15)	0.8616	0.8631	0.8602
	Full alphabet (10)	0.8589	0.8603	0.8574
	Full alphabet (6)	0.8416	0.8432	0.8400
MCC	PASTA 2.0	0.4296	0.4332	0.4261
	FoldAmyloid	0.4531	0.4566	0.4497
	appnn	0.5817	0.5847	0.5787
	AmyloGram (15)	0.5420	0.5446	0.5394
	AmyloGram (10)	0.6300	0.6330	0.6271
	AmyloGram (6)	0.6037	0.6069	0.6004
	Full alphabet (15)	0.5488	0.5519	0.5456
	Full alphabet (10)	0.5716	0.5749	0.5683
	Full alphabet (6)	0.5436	0.5467	0.5404
Sensitivity	PASTA 2.0	0.3833	0.3863	0.3803
	FoldAmyloid	0.7518	0.7545	0.7490
	appnn	0.8864	0.8885	0.8843
	AmyloGram (15)	0.9463	0.9477	0.9448
	AmyloGram (10)	0.8647	0.8669	0.8626
	AmyloGram (6)	0.6770	0.6800	0.6741
	Full alphabet (15)	0.8175	0.8200	0.8151
	Full alphabet (10)	0.7521	0.7549	0.7493
	Full alphabet (6)	0.4984	0.5016	0.4952
Specificity	PASTA 2.0	0.9519	0.9530	0.9509
	FoldAmyloid	0.7190	0.7211	0.7168
	appnn	0.7211	0.7232	0.7190
	AmyloGram (15)	0.6113	0.6136	0.6090
	AmyloGram (10)	0.7890	0.7910	0.7871
	AmyloGram (6)	0.9028	0.9042	0.9014
	Full alphabet (15)	0.7527	0.7548	0.7506
	Full alphabet (10)	0.8271	0.8290	0.8252
	Full alphabet (6)	0.9591	0.9600	0.9582

S5 Pairwise sequence identity between training and benchmark data sets

For each peptide from the *pep424* data set we computed its pairwise identity to peptides from the benchmark data set. The pairwise sequence identity was defined as following (Raghava and Barton, 2006):

$$\text{Pairwise identity} = \frac{I}{A+G}. \tag{1}$$

where:

- I: identical positions,
- A: aligned positions,
- G: internal gap positions.

We discovered that despite high pairwise identity, peptides may have different properties amyloidogenic properties. In case of 270 non-amyloidogenic sequences from *pep424*, over 295 amyloidogenic peptides from the training data set have pairwise identity 100%. 149 amyloidogenic peptides from the *pep424* data set have pairwise identity 100% with only 69 sequences in the benchmark data set that are amyloidogenic and 316 non-amyloidogenic sequences (2). Concluding, in case of amyloid data high sequence similarity does not reflect likeness in their properties.

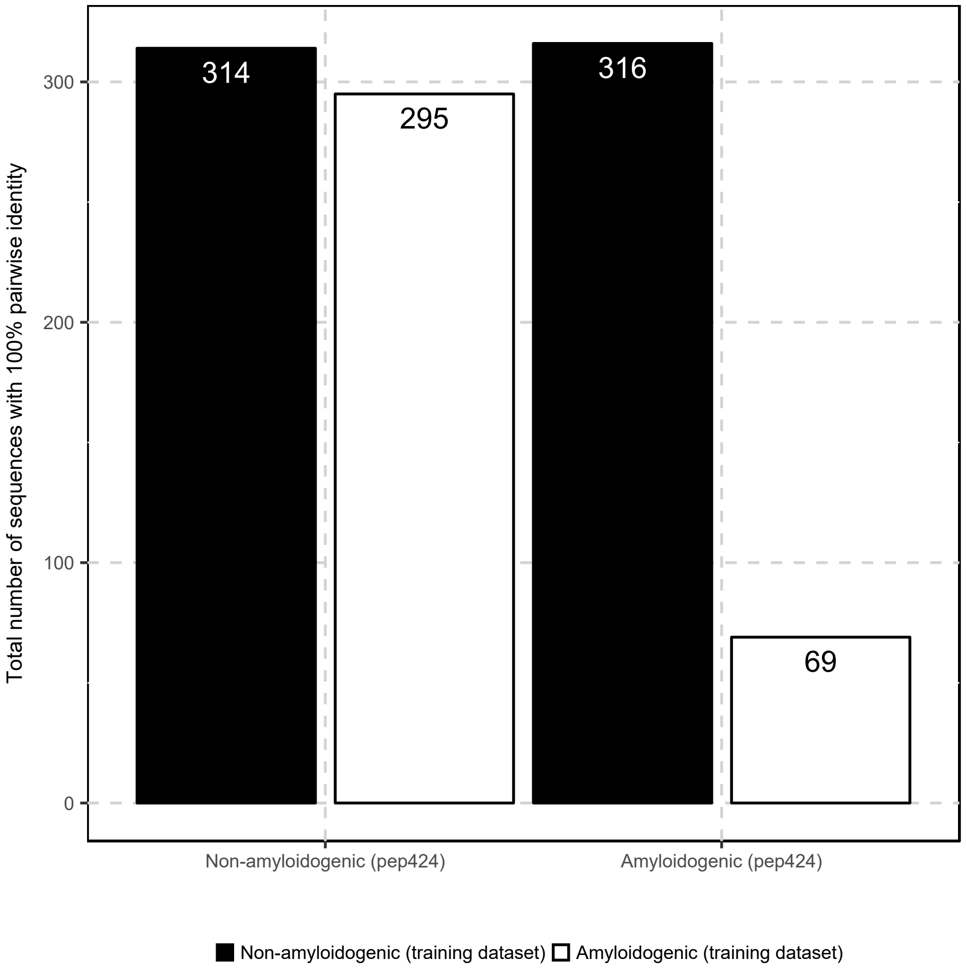

Figure 2: Number of sequences from the benchmark data set with pairwise identity 100% with sequences from the *pep424* data set.

S6 Jackknife test

To further estimate the bias of AmyloGram, we performed a jackknife procedure. Using the training data created for the benchmark (269 positive sequences and 746 negative sequences) we trained 961 iterations of AmyloGram each time leaving one sequence out and then performing predictions on the left sequence. The AUC=0.86 and MCC=0.52. We also trained an iteration of AmyloGram on the full training data set and made predictions on all input sequences obtaining AUC=0.97 and MCC=0.80.

S7 Amino acid flexibility/rigidity and size

In the light of recent studies, amyloid peptides could form ring-like structures in the core of aggregating oligomers Dovidchenko *et al.* (2016). This may require a certain flexibility of amino acids involved in the core. However, the flexibility/rigidity seems to depend on the size or volume of amino acid residues. Therefore, we checked if the flexibility measure that stood out in our analysis of amyloid regions can result from a correlation to any size-related feature of amino acids, especially that which was not selected for the encodings. We evaluated the correlation of three size-related properties from AAIndex database and the flexibility measure chosen by our algorithm (Tab. 4 and Fig. 3). It turned out that only bulkiness is significantly correlated but moderately with the amino acid flexibility (Tab. 4). Bulkiness was not in the set of 17 physicochemical properties used to create the encodings.

Table 4: The correlation coefficient and its significance between the flexibility measure and three parameters describing the size of amino acids.

	Parameter	Pearson's correlation coefficient	p-value
1	Size (Dawson, 1972)	-0.27	0.24
2	Residue volume (Bigelow, 1967)	-0.42	0.06
3	Bulkiness (Zimmerman et al., 1968)	-0.49	0.03

We also computed the average values of flexibility and size-related measures of each peptide from the AmyLoad database. The average size is very similar for amyloid and non-amyloid peptides, as well as is not related to the visible differences in the flexibility. It can be seen on volcano plots, where the distribution of flexibility differentiates the amyloid and non-amyloid peptides (Fig. 5)). The same can be also observed on violin plots representing the distribution of mean values of all properties for amyloids and non-amyloids (Fig. 4)). A slight difference can be observed in the volume of residues, however this feature was explicitly selected for the encodings and is not correlated with the flexibility measure that we used. Finally, the bulkiness behaves somehow differently. It may differentiate amyloid and non-amyloid hot-spots and it is correlated with our flexibility measure. Therefore, we cannot exclude that this size-related measure may contribute to the amyloid propensity.

S8 Bibliography

Argos, P., Rao, J. K., and Hargrave, P. A. (1982). Structural prediction of membrane-bound proteins. *European journal of biochemistry / FEBS*, **128**(2-3), 565–575.

Bhaskaran, R. and Ponnuswamy, P. (1988). Positional flexibilities of amino acid residues in globular proteins. *International Journal of Peptide and Protein Research*, **32**(4), 241–255.

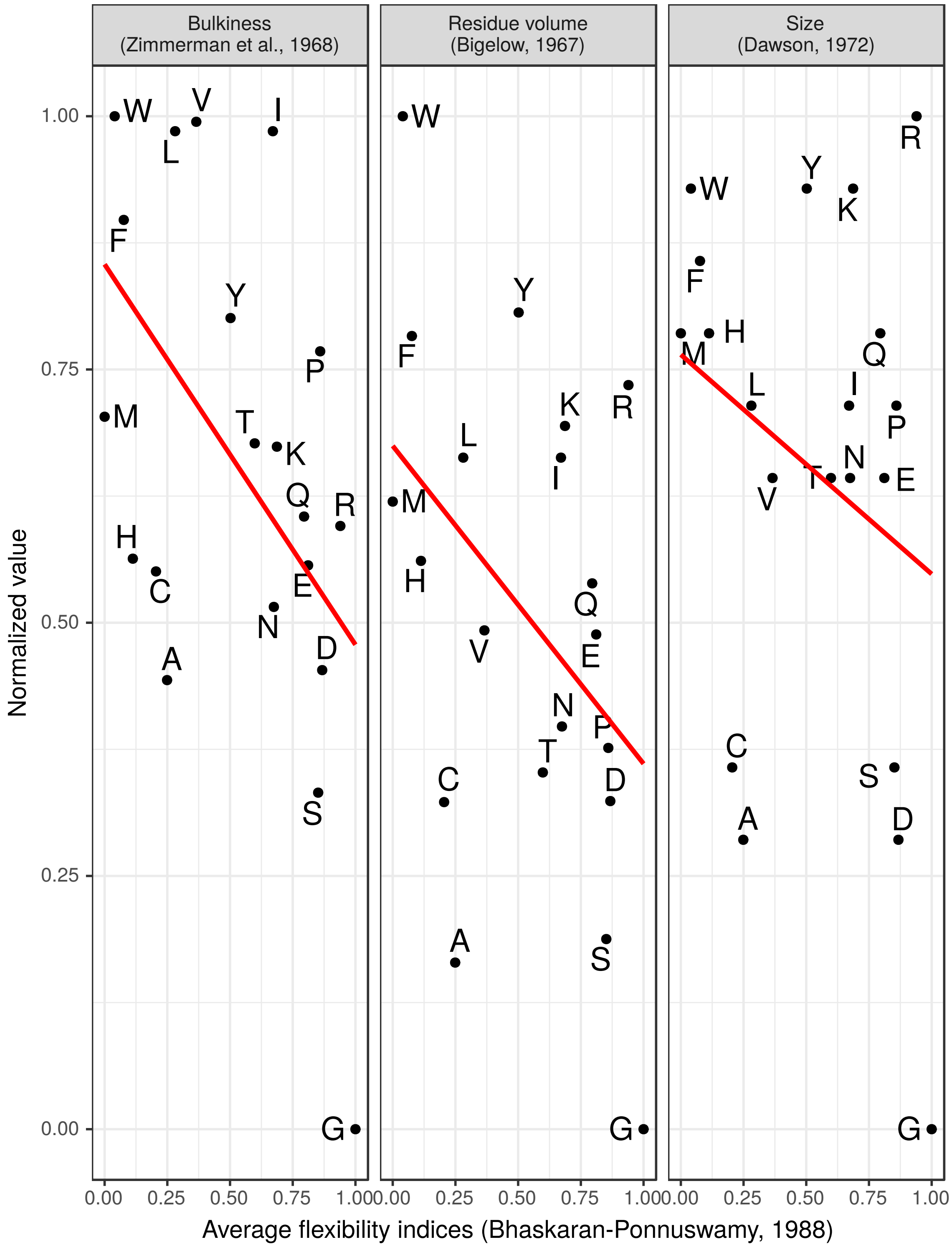

Figure 3: Correlations between the average flexibility indices and the size-related measures.

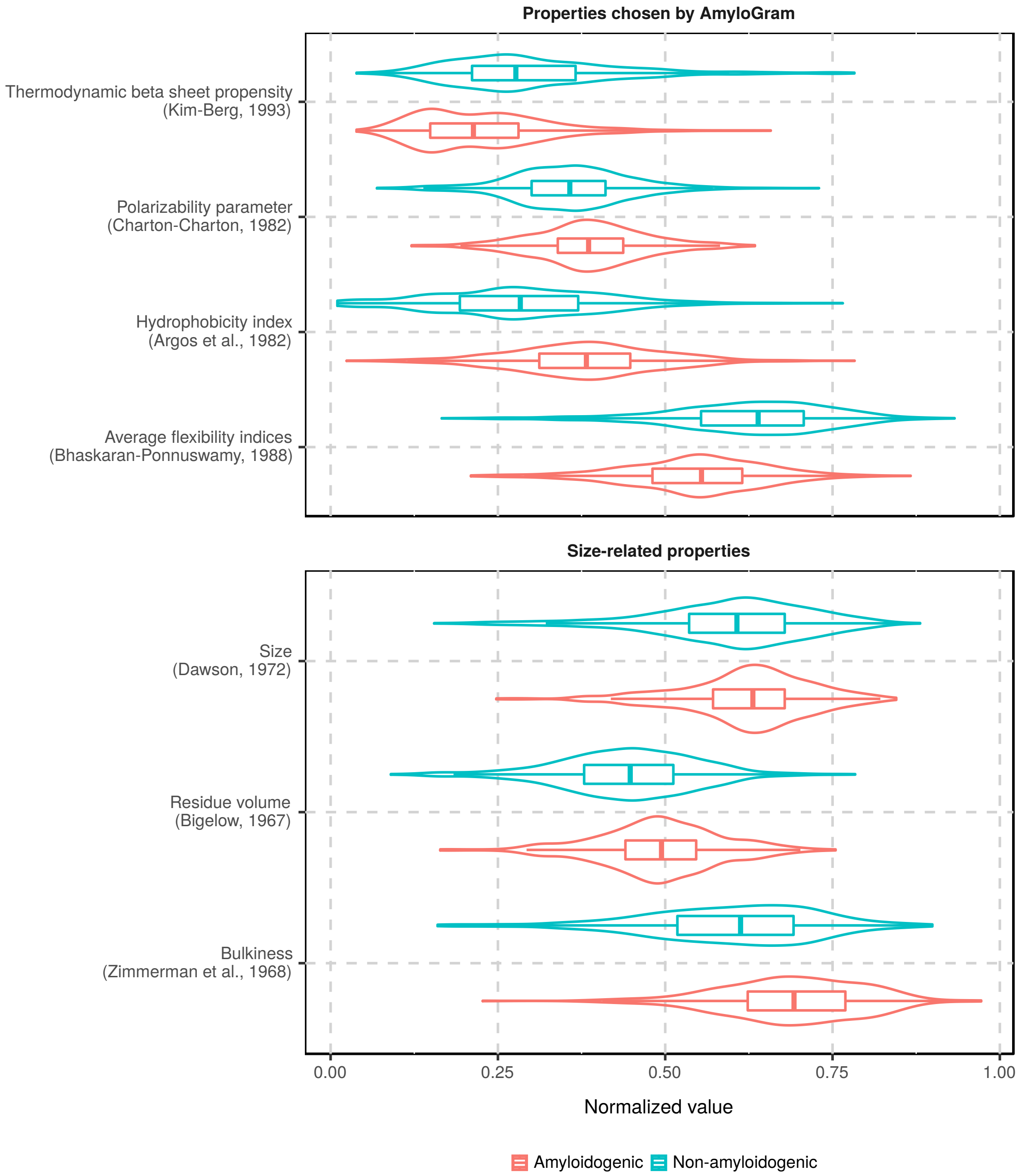

Figure 4: Density distribution of all analyzed properties.

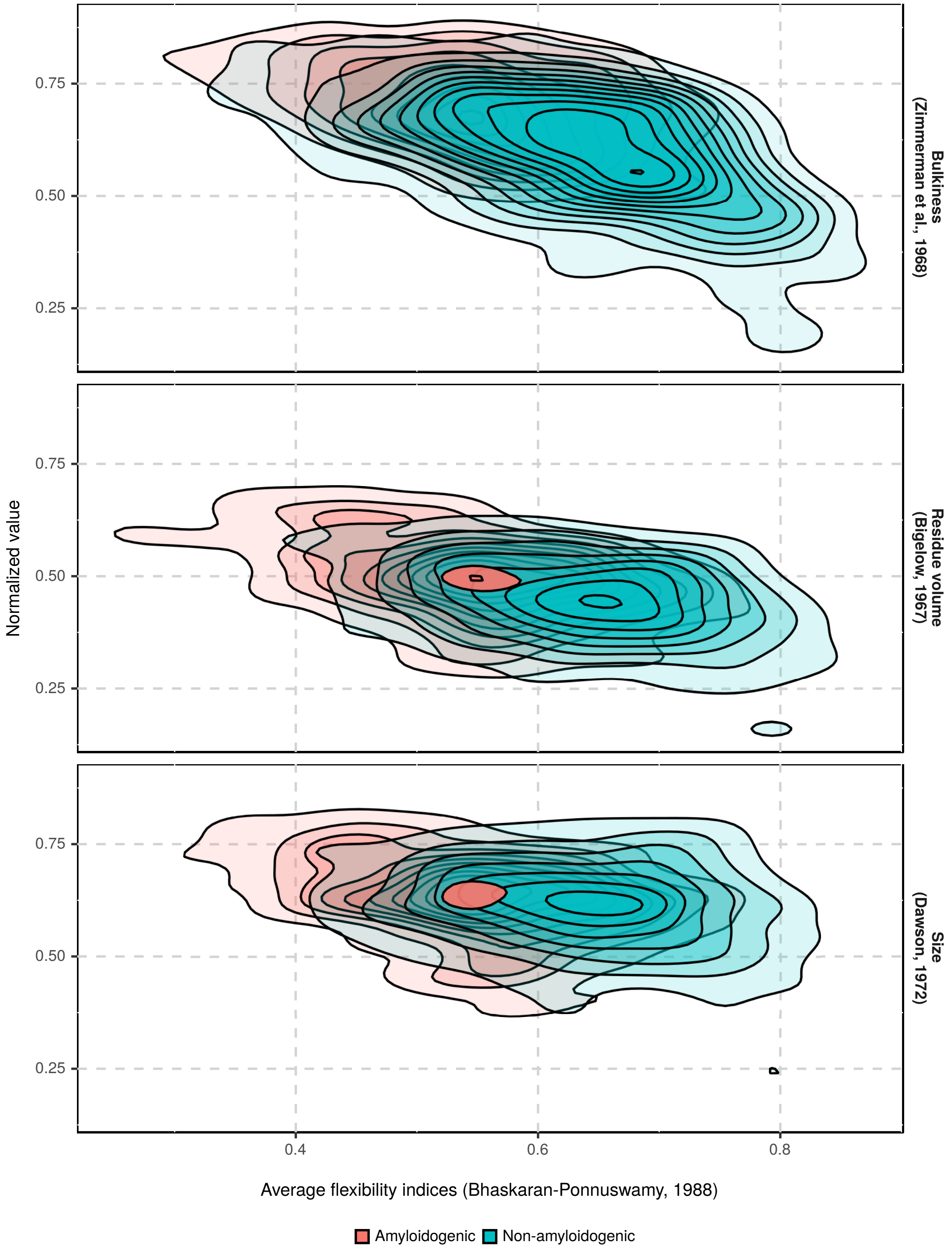

Figure 5: Density plots of size-related parameters and flexibility for peptides collected in AmyLoad database.

Black, S. D. and Mould, D. R. (1991). Development of hydrophobicity parameters to analyze proteins which bear post- or cotranslational modifications. *Analytical Biochemistry*, **193**(1), 72–82.

Charton, M. and Charton, B. I. (1982). The structural dependence of amino acid hydrophobicity parameters. *Journal of Theoretical Biology*, **99**(4), 629–644.

Dovidchenko, N. V., Glyakina, A. V., Selivanova, O. M., Grigorashvili, E. I., Suvorina, M. Y., Dzhus, U. F., Mikhailina, A. O., Shiliaev, N. G., Marchenkov, V. V., Surin, A. K., and Galzitskaya, O. V. (2016). One of the possible mechanisms of amyloid fibrils formation based on the sizes of primary and secondary folding nuclei of AB40 and AB42. *Journal of Structural Biology*, **194**(3), 404–414.

Efron, B. and Tibshirani, R. J. (1994). *An Introduction to the Bootstrap*. CRC Press. Google-Books-ID: gLlpIUxRntoC.

Kanehisa, M. I. and Tsong, T. Y. (1980). Local hydrophobicity stabilizes secondary structures in proteins. *Biopolymers*, **19**(9), 1617–1628.

Kidera, A., Konishi, Y., Oka, M., Ooi, T., and Scheraga, H. A. (1985). Statistical analysis of the physical properties of the 20 naturally occurring amino acids. *Journal of Protein Chemistry*, **4**(1), 23–55.

Kim, C. A. and Berg, J. M. (1993). Thermodynamic beta-sheet propensities measured using a zinc-finger host peptide. *Nature*, **362**(6417), 267–270.

Kosiol, C., Goldman, N., and Buttimore, N. H. (2004). A new criterion and method for amino acid classification. *Journal of Theoretical Biology*, **228**(1), 97–106.

Lehmann, E. L. and Romano, J. P. (2008). *Testing Statistical Hypotheses*. Springer New York.

Melo, F. and Marti-Renom, M. A. (2006). Accuracy of sequence alignment and fold assessment using reduced amino acid alphabets. *Proteins*, **63**(4), 986–995.

Nishikawa, K. and Ooi, T. (1986). Radial locations of amino acid residues in a globular protein: correlation with the sequence. *Journal of Biochemistry*, **100**(4), 1043–1047.

Pontius, J., Richelle, J., and Wodak, S. J. (1996). Deviations from Standard Atomic Volumes as a Quality Measure for Protein Crystal Structures. *Journal of Molecular Biology*, **264**(1), 121–136.

Prabhakaran, M. (1990). The distribution of physical, chemical and conformational properties in signal and nascent peptides. *The Biochemical Journal*, **269**(3), 691–696.

Radzicka, A. and Wolfenden, R. (1988). Comparing the polarities of the amino acids: side-chain distribution coefficients between the vapor phase, cyclohexane, 1-octanol, and neutral aqueous solution. *Biochemistry*, **27**(5), 1664–1670.

Raghava, G. and Barton, G. J. (2006). Quantification of the variation in percentage identity for protein sequence alignments. *BMC Bioinformatics*, **7**, 415.

Sweet, R. M. and Eisenberg, D. (1983). Correlation of sequence hydrophobicities measures similarity in three-dimensional protein structure. *Journal of Molecular Biology*, **171**(4), 479–488.

Takano, K. and Yutani, K. (2001). A new scale for side-chain contribution to protein stability based on the empirical stability analysis of mutant proteins. *Protein Engineering*, **14**(8), 525–528.

Wozniak, P. P. and Kotulska, M. (2014). Characteristics of protein residue-residue contacts and their application in contact prediction. *Journal of Molecular Modeling*, **20**(11).

Zhou, H. and Zhou, Y. (2004). Quantifying the effect of burial of amino acid residues on protein stability. *Proteins*, **54**(2), 315–322.
